# Regulation of Aerobic Energy Metabolism in *Podospora anserina* by Two Paralogous Genes Encoding Structurally Different *c*-Subunits of ATP Synthase

**DOI:** 10.1371/journal.pgen.1006161

**Published:** 2016-07-21

**Authors:** Carole H. Sellem, Jean-Paul di Rago, Jean-Paul Lasserre, Sharon H. Ackerman, Annie Sainsard-Chanet

**Affiliations:** 1 Institute for Integrative Biology of the Cell (I2BC), CEA-CNRS-Université Paris-Sud, Gif sur Yvette, France; 2 CNRS, Institut de Biochimie et Génétique Cellulaires, UMR 5095, Bordeaux, France; 3 Université Victor Segalen-Bordeaux 2, IBGC, UMR 5095, Bordeaux, France; 4 Department of Biochemistry and Molecular Biology, Wayne State University School of Medicine, Detroit, Michigan, United States of America; Max Planck Institute for Biology of Ageing, GERMANY

## Abstract

Most of the ATP in living cells is produced by an F-type ATP synthase. This enzyme uses the energy of a transmembrane electrochemical proton gradient to synthesize ATP from ADP and inorganic phosphate. Proton movements across the membrane domain (F_O_) of the ATP synthase drive the rotation of a ring of 8–15 *c*-subunits, which induces conformational changes in the catalytic part (F_1_) of the enzyme that ultimately promote ATP synthesis. Two paralogous nuclear genes, called *Atp9-5* and *Atp9-7*, encode structurally different *c*-subunits in the filamentous fungus *Podospora anserina*. We have in this study identified differences in the expression pattern for the two genes that correlate with the mitotic activity of cells in vegetative mycelia: *Atp9-7* is transcriptionally active in non-proliferating (stationary) cells while *Atp9-5* is expressed in the cells at the extremity (apex) of filaments that divide and are responsible for mycelium growth. When active, the *Atp9-5* gene sustains a much higher rate of *c*-subunit synthesis than *Atp9-7*. We further show that the ATP9-7 and ATP9-5 proteins have antagonist effects on the longevity of *P*. *anserina*. Finally, we provide evidence that the ATP9-5 protein sustains a higher rate of mitochondrial ATP synthesis and yield in ATP molecules *per* electron transferred to oxygen than the *c*-subunit encoded by *Atp9-7*. These findings reveal that the *c*-subunit genes play a key role in the modulation of ATP synthase production and activity along the life cycle of *P*. *anserina*. Such a degree of sophistication for regulating aerobic energy metabolism has not been described before.

## Author Summary

In mitochondria, the ATP synthase (also referred to as complex V) catalyzes the late steps of oxidative phosphorylation (OXPHOS), which is a process that provides aerobic eukaryotes with most of their energy requirements by generating adenosine triphosphate (ATP) molecules. While the structure and mechanism of ATP synthase are mostly well established, much remains to be learned about how cells and tissues modulate the production and activity of this enzyme. Herein we report the existence in the filamentous fungus *Podospora anserina* of a two-pronged energy regulatory mechanism that involves two nuclear genes (*Atp9-5 and Atp9-7*) that encode structurally different *c*-subunits of ATP synthase. This system enables a proper production of ATP synthase and optimizes the rate of ATP synthesis in mitochondria along the rather complex life cycle of this fungus.

## Introduction

Most energy-transducing membranes in bacteria, mitochondria and chloroplasts contain an F-ATP synthase. Historically this enzyme has been described in terms of an integral membrane domain (F_O_) and a peripheral domain (F_1_). High-definition structural data now available reveal that F_O_ and F_1_ are further divided into distinct oligomeric sub-structures [1–4]. The most hydrophobic F_O_ subunits associate in a unit, composed of one *a*-subunit adjacent to a ring of 8–15 *c*-subunits, that transfers protons from one side of the membrane to the other. The remaining F_O_ subunits assemble a stalk contacting at one end the *a*-subunit in the membrane and attached at the other end to the external periphery of the soluble F_1_ domain. F_1_ is made of a globular structure ([αβ]_3_ hexamer) that contains three catalytic sites, one in each β-subunit, and 2–3 other proteins that constitute a central stalk connecting these sites at one end with the *c*-ring at the other end. Energy released by proton translocation through the F_O_ activates the rotation of the *c*-ring together with the central stalk relative to the non-moving parts of ATP synthase, which forces conformational changes in the catalytic sites that facilitate product (ATP) release from the enzyme [5].

The proton channel in F-ATP synthases extends along a proteinacious interface that is formed by transmembrane a-helices of subunits *a* and *c*. The individual monomers of the *c*-ring adopt a hairpin structure, composed of two membrane-spanning α-helices (numbered 1 and 2) joined by a short hydrophilic linker. In cross section, the *c*-ring shows a double annular structure with inner and outer circles comprised of helices 1 and 2, respectively. According to this arrangement, only helix 2 contributes to the proton channel. A recently proposed model of the *a*/*c*-ring complex defines the proton channel by α-helices 4 and 5 in the *a*-subunit and helix 2 of the *c*-subunit [6]. The most notable feature of this domain is a universally conserved acidic amino acid (Glu or Asp) in *c*-subunit that participates directly in proton transfer. The acidic residue is located in the middle of helix 2 where it would project into the hydrophobic phase of the bilayer around the perimeter of the *c*-ring. Hence, its carboxyl group is assumed to be protonated in all of the *c*-subunits except for the monomer that is located at the *a*/*c*-ring interface. Here, the hydrophilic environment inside the ion channel would favor the ionized carboxylate. In the proposed series of events that occur during proton translocation the carboxylate group inside the channel is neutralized with a proton that originates from the *p*-side of the membrane, followed by rotation of the *c*-ring, which moves the newly protonated *c*-subunit to the lipid phase and the adjacent monomer into the channel. Deprotonation of the incoming carboxyl group transfers a proton to the channel, and ultimately to the *n*-side of the membrane to complete the process of uni-directional proton translocation.

Previous work revealed the existence in the filamentous fungus *Podospora anserina* of two separate nuclear genetic loci (*Atp9-5* and *Atp9-7*) that encode two different *c*-subunit isoforms that share 66% sequence identity (in their mature part) [7]. Work by others has revealed the presence of multiple isogenes encoding the *c*-subunit also in the nuclear genomes of mammals [8,9] and the parasite *Trypanosoma brucei* [10]. All three mammalian isoforms are identical beyond the mitochondrial leader cleavage site, whereas those from *T*. *brucei* show between them (after the predicted processing site) sequence variation in the first 20% of the primary sequence. All three isoforms are produced and assembled in the *T*. *brucei* enzyme during the different life stages of the parasite, though it remains to be determined if individual enzyme molecules are mosaic with respect to the *c*-subunit or if each isoform preferentially assembles with the same type such that there are different populations of the F-ATP synthase in the cell. The *c*-subunits in *P*. *anserina* are distinguished on the basis that the sequence variation is distributed throughout the entire length of the mature proteins.

The vegetative phase of *P*. *anserina* is initiated by ascospore germination, which gives rise to a network (thallus) of branched filaments or hyphae that spread out to form a mycelium. Unlike plant and animal cells, those of filamentous fungi form a continuous multi-nucleated cytoplasm, the growth of which is restricted to the tip (apex) of hyphae (polarized growth). For simplification, “proliferating or apical cells” will be used henceforth to designate the growing apex, while “non proliferating cells” will designate the non-growing part of hyphae backwards the apex. The current paper shows that in vegetative cultures of *P*. *anserina*, *Atp9-5* and *Atp9-7* are expressed in different locations; the former at the apex of hyphae and the latter in non-proliferating cells that comprise the bulk of the fungal mycelium. We also show that the proteins encoded by *Atp9-5* and *Atp9-7* (referred to as ATP9-5 and ATP9-7 respectively) have antagonist effects on the longevity of *P*. *anserina* and confer to the mitochondria different bioenergetics properties. These findings reveal that the two *c*-subunit genes play a crucial role in the modulation of mitochondrial energy transduction along the life cycle of this filamentous fungus.

## Results

### Transcription profiling of *Atp9-7* and *Atp9-5* in vegetative cultures of *P*. *anserina*

**Fig 1:**
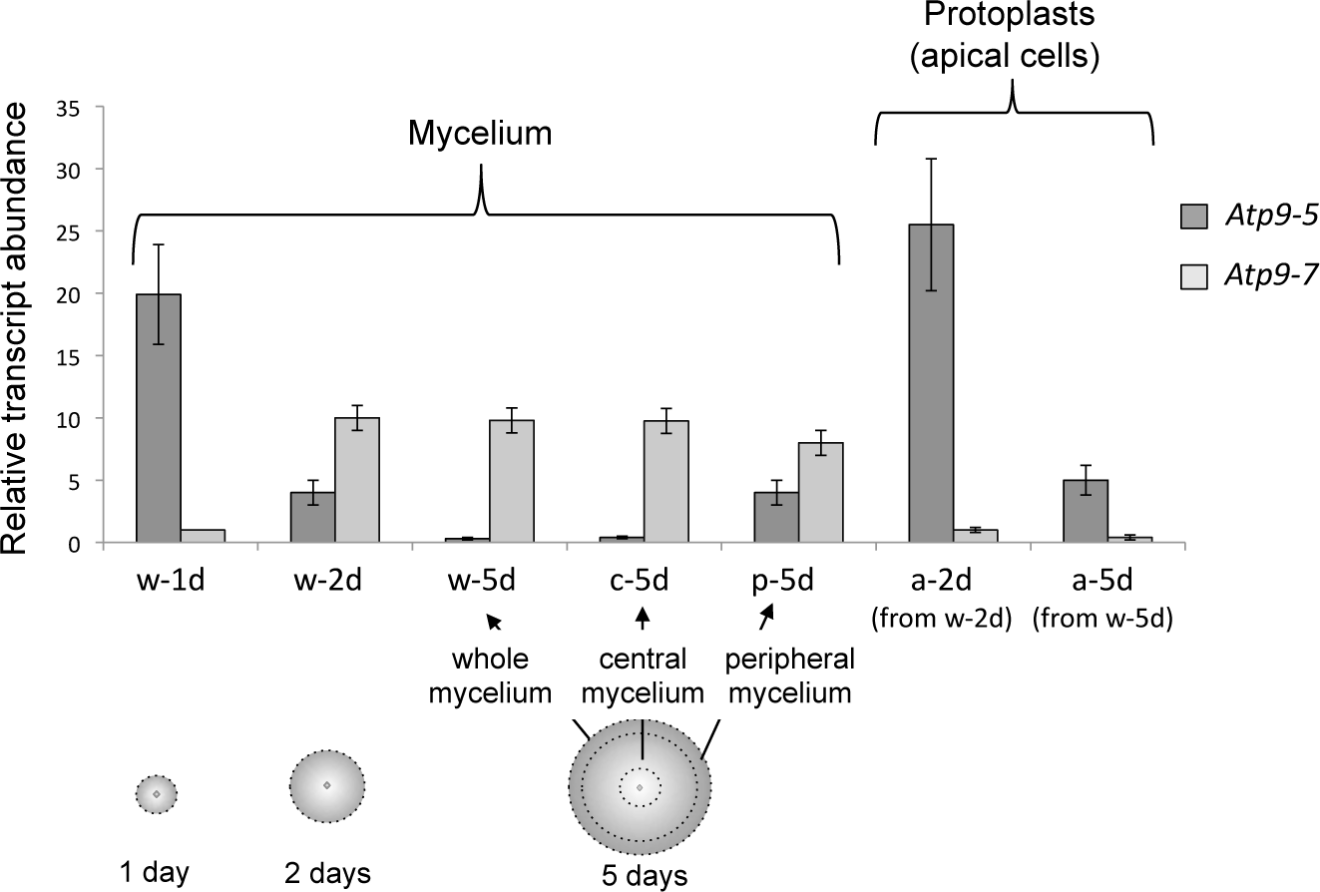
Transcription profiling of *Atp9-7* and *Atp9-5* in vegetative cultures of *P*. *anserina*. The relative abundance of *Atp9-5* and *Atp9-7* mRNA transcripts in vegetative cultures of *P*. *anserina*, *versus* a constitutively expressed gene (*Gpd*), was determined by real-time quantitative reverse transcription PCR. The analyzed RNA extracts were prepared from: (i) whole cultures grown on solid medium for 1 (w-1d), 2 (w-2d) and 5 (w-5d) days; (ii) the central (c-5d) and peripheral (p-5d) regions of w-5d mycelium as delineated by dotted lines; and (iii) protoplasts (apical cells) obtained from 2 (a-2d) and 5 days (a-5d) aged liquid cultures. The results are presented as histograms with an arbitrary value of 1 for *Atp9-7* transcripts in w-1d. The error bars indicate standard error (SEM) in at least three independent experiments.

**Fig 2:**
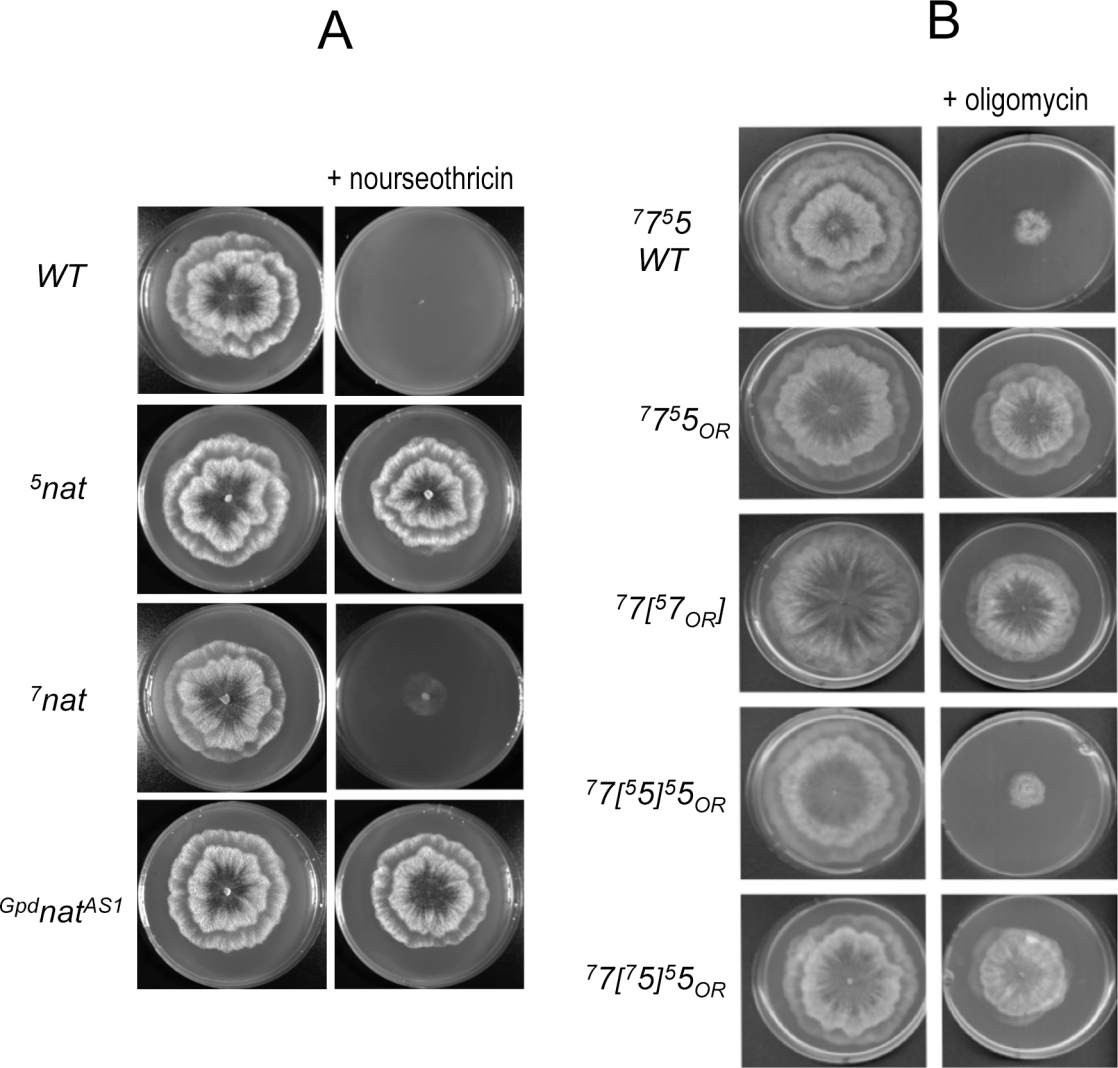
Expression of inhibitor-resistance genes from the *Atp9-7* and *Atp9-5* regulatory sequences. (A) Expression of *nat1*, a nourseothricin-resistance conferring gene. The wild type of *P*. *anserina* (*WT*) does not contain any *nat1* transgene gene and consequently fails to grow in the presence of nourseothricin (M2 plates supplemented with 50 μg/ml of the drug) owing to a block in protein synthesis. Strain ^*5*^*nat* harbors a *nat1* gene under control of the 5’ and 3’ *cis*-regulatory sequences of *Atp9-5*. In strain ^*7*^*nat*, the nourseothricin-resistance gene is controlled by the regulatory sequences of *Atp9-7*. The control inhibitor-resistant strain (^*Gpd*^*nat*^*AS1*^) carries a *nat1* transgene with the 5’ flanking sequence from *Gpd* and the 3’ flanking sequence from *AS1*, both of which conferred a high level of constitutive expression [12]. The plates were photographed after 4 days of incubation. (B) Expression of oligomycin-resistance alleles in *Atp9-5* (F124S) and *Atp9-7* (F135Y). In the short-hand nomenclature used to distinguish the various strains, the numbers *5* and *7* in regular point size indicate the *Atp9-5* and *Atp9-7* alleles, respectively, while the origin of regulatory sequences upstream and downstream the *Atp9* reading frame is indicated with a superscript number (similar to that described in panel A with the *nat1* gene); the subscript “*O*_*R*_” denotes a mutant allele that confers oligomycin-resistance; and ectopic genes are surrounded by brackets. Resistance to oligomycin was tested on M2 plates supplemented with 0.5 μg/ml of the drug. The plates were photographed after 5 days of incubation.

The relative abundance of mRNA transcripts from *Atp9-5* and *Atp9-7*, *versus* a constitutively expressed gene (*Gpd*), was determined by real-time quantitative reverse transcription PCR as described under Materials and Methods. RNA extracts were prepared from whole mycelium cultured on solid media for 1 day (w-1d), 2 days (w-2d), or 5 days (w-5d). *Atp9-5* transcripts were 20-fold more abundant than *Atp9-7* transcripts in w-1d RNA samples (Fig 1, w-1d). With time, the level of *Atp9-5* transcripts declined to near zero while *Atp9-7* transcripts rose rapidly, reaching a steady state by day 2 (Fig 1, w-2d, w-5d). These results suggested that *Atp9-5* and *Atp9-7* are expressed differentially in proliferating and non-proliferating cells, respectively. The apex of hyphae establishes a zone of proliferation around the perimeter of the discoidal mycelium in plate cultures of *P*. *anserina*. These mitotically active cells comprise a significant percentage of total cells in 1-day-old mycelium, but they are far surpassed in number by non-dividing cells that accumulate during radial expansion of the mycelium. As such, any transcript unique to apical cells (*e*.*g*. *Atp9-5*) would be a minor species in the total RNA extracted from whole preparations of 5-day-old mycelium, and difficult to detect (Fig 1, w-5d).

Our hypothesis was confirmed in experiments that targeted cells isolated from different regions of mycelium. Non-proliferating cells collected from the center of 5-day-old mycelium were abundant in *Atp9-7* transcripts and almost completely deficient for *Atp9-5* transcripts (Fig 1, c-5d). Instead, peripheral samples collected from the same mycelium (Fig 1, p-5d), enriched for apical cells, contained a significant amount of *Atp9-5* transcripts, but it was not clear if the *Atp9-7* transcripts that were co-detected originated from mitotically active apical cells or from stationary cells present in the peripheral sample. Therefore, protoplasts derived from apical cells of 2-day-old and 5-day-old mycelia were isolated and used to prepare RNA samples that better reflected the transcriptional activity unique to proliferating cells. In the end we found that *Atp9-5* transcripts clearly dominated *Atp9-7* transcripts in these cells (Fig 1 a-2d, a-5d).

Cumulatively, these results lead us to propose that the origin of *c*-subunit mRNA in *P*. *anserina* is dictated by the mitotic status of the cells. It is also noteworthy that the levels of *Atp9-7* transcripts in w-2d and w-5d were much lower compared to those of *Atp9-5* in w-1d. Hence it would appear that the transcriptional switch from *Atp9-5* in proliferative cells to *Atp9-7* in non-proliferative cells is accompanied by a considerable decrease in the rate of *c*-subunit synthesis.

### Investigation of *Atp9-5* and *Atp9-7* regulatory sequences

Unfortunately, we failed to raise antibodies that specifically recognized the ATP9-5 and ATP9-7 proteins, which could have been especially useful to determine directly how these proteins are expressed along the life cycle of P. anserina. Furthermore, while these proteins can functionally substitute for yeast subunit 9 [11] adding tags to them severely compromises ATP synthase function (S1 Fig). The use of fluorescence protein markers, like GFP, under control of the regulatory sequences of *Atp9-7* and *Atp9-5* did not seem to us a good alternative too. Indeed, though the transcripts data indicate that ATP9-5 is preferentially, if not exclusively, synthesized in proliferating cells, this does not mean that it will not be present in non-proliferating cell. As a result, and considering the well-known stability of GFP, this protein will likely distribute throughout the entire mycelium even if it is exclusively synthesized at the apex.

As an alternative approach, we took advantage of the sensitivities of *P*. *anserina* to some chemical inhibitors to evaluate *cis*-regulatory sequences in *Atp9-5* and *Atp9-7* for the initiation of growth and the continued propagation of fungal mycelium on selective media. Since growth occurs only at the apex of filaments (see above), any gene that is required to confer resistance to the inhibitor must be actively transcribed in apical cells. Instead, if the required gene is silent or transcribed too poorly at the apex to provide a sufficient amount of the necessary protein, the mycelium will retain inhibitor sensitivity and show a growth defect on the inhibitor-containing plates.

#### Studies with the protein synthesis inhibitor nourseothricin

The *nat1* gene encodes an N-acetyl transferase that inactivates nourseothricin, a natural antibiotic that inhibits protein synthesis in a wide range of organisms including *P*. *anserina* [12]. Versions of the *nat1* gene under the control of the 5’ and 3’ flanking sequences of *Atp9-5* (referred to as ^*5*^*nat* in which the superscript denotes the sequences flanking *Atp9-5*) or *Atp9-7* (^*7*^*nat*) were constructed and randomly co-integrated with a phleomycin-resistance gene at various chromosomal positions in the wild type strain (see Supporting Information for strain construction and S1 Table for complete genotype). We isolated ~30 independent phleomycin-resistant transformants for each transgene and monitored their growth on plates in the absence or presence of nourseothricin (Fig 2A, results shown only for one representative ^*5*^*nat* and ^*7*^*nat* clone; see S2 Table for details). The control strain carried a *nat1* transgene (^*Gpd*^*nat*^*AS1*^) with the 5’ flanking sequence from *Gpd* and the 3’ flanking sequence from *AS1*, both of which confer a high level of constitutive expression [12]. The *AS1* gene encodes a cytosolic ribosomal protein (S12) that was identified by a mutation showing *a*nti-*s*uppressor activity of translational fidelity defects [13,14]. Strains carrying either the ^*5*^*nat* or ^*Gpd*^*nat*^*AS1*^ transgene grew robustly in the presence of nourseothricin in contrast to ^*7*^*nat* strains (Fig 2A, S2 Table). In accordance with previous work showing that of the two *Atp9* genes only *Atp9-5* is actively transcribed during spore germination [7] we also determined that ^*5*^*nat* spores, but not ^*7*^*nat* spores, could germinate in the presence of nourseothricin (S2 Table). These findings suggest there is some aspect of the regulatory sequences in *Atp9-5* that is unique with respect to *Atp9-7*, which activates *nat1* in apical cells and allows the fungus to grow/germinate on media supplemented with the drug.

#### Studies with the ATP synthase inhibitor oligomycin

The antibiotic oligomycin is a well-characterized, specific inhibitor of mitochondrial ATP synthase that prevents proton translocation through its integral membrane ion channel. A high resolution X-ray structure of the enzyme from *Saccharomyces cerevisiae* has revealed that oligomycin binds to the surface of the *c*-ring near the middle of helix 2 in two adjacent monomers [15]. In doing so the inhibitor blocks access to the critical acidic residue (Glu59 in yeast) that serves as the actual proton carrier in the reaction. Previous work has provided multiple examples in which yeast were conferred an oligomycin-resistant (O_R_) phenotype by the substitution of a single amino acid in the *c*-subunit [16–18]. We exploited this characteristic and developed a novel selection assay for the expression of *Atp9-5* or *Atp9-7* in apical cells. As will be described next we isolated *P*. *anserina* mutants that harbor a single site missense mutation in either *Atp9-5* or *Atp9-7* and determined under what conditions one or the other mutant *O*_*R*_ allele was expressed at the apex at a level sufficient to permit the growth of mycelium on oligomycin plates. However, before proceeding it is important to first explain the short-hand nomenclature that will be used from this point forward to distinguish fungal strains that have different genetic backgrounds with respect to *Atp9*: (i) the numbers *5* and *7* shown in regular point size indicate the *Atp9-5* and *Atp9-7* alleles, respectively; (ii) the origin of regulatory sequences upstream and downstream the *Atp9* reading frame is indicated with a superscript number (similar to that described above with the *nat1* gene); (iii) the subscript “*OR*” is used to denote a mutant allele of *Atp9* that confers oligomycin-resistance to the ATP synthase; (iv) brackets ([]) are used to distinguish ectopic versions of the *Atp9-5* and *Atp9-7* genes, *i*.*e*. when located at a locus distinct from the one they normally occupy; and (vi) null alleles of the native *Atp9-7* and *Atp9-5* (referred to *∆7* and *∆5* in S1 Table) are not mentioned in the short-hand nomenclature of strains (see Supporting information for a complete genotype of strains (S1 Table) and how they were obtained). Two examples: (i) strain ^*7*^*7[*^*5*^*5]*^*5*^*5*_*OR*_ contains the native *Atp9-7* gene (^*7*^*7*), an ectopic version of the *Atp9-5* gene under control of its native regulatory sequence ([^*5*^*5*]), and an oligomycin-resistance allele in the native *Atp9-*5 gene (^*5*^*5*_*OR*_); strain *[*^*7*^*5][*^*5*^*7]* is deleted for both the native *Atp9-7* and *Atp9-5* genes, contains an ectopic *Atp9-5* gene under control of the regulatory sequences of *Atp9-7* ([^*7*^*5*]), and an ectopic *Atp9-7* gene under control of the regulatory sequences of *Atp9-5* ([^*5*^*7*]).

Wild type *P*. *anserina* was exposed to ultraviolet radiation (see under Materials and Methods) and screened for growth in the presence of oligomycin to select *O*_*R*_ strains that harbored a mutant allele at one of the two genetic loci coding for the *c*-subunit. For this to occur, there must be expression of an oligomycin-resistant allele of *Atp9* at the apex of the filaments. Three independent *O*_*R*_ isolates, each one carrying the identical mutation (F124S) in *Atp9-5*, were isolated. In contrast to wild type strain (designated ^*7*^*7*^*5*^*5*), a representative mutant strain (^*7*^*7*^*5*^*5*_*OR*_) grew quite well on oligomycin plates (Fig 2B; see also S3 Table). Back crosses to the wild-type strain confirmed that the F124S mutation was alone responsible for the drug resistance phenotype of these strains. Indeed, *O*_*R*_ segregated as a nuclear monogenic marker, and the mutated allele was always identified by DNA sequencing in monokaryotic *O*_*R*_ progenies, never in the drug sensitive progenies.

To reconcile the fact that none of the *O*_*R*_ strains isolated from the wild type bore a mutation linked to *Atp9-7*, we reasoned that this locus was too poorly transcribed in the apical cells of the wild type to provide a sufficient amount of mutant *c*-subunit resistant to the drug. This idea prompted us to construct a chimeric gene in which regulatory sequences from *Atp9-5* flank the *Atp9-7* reading frame (identified by the notation *[*^*5*^*7]* in the strain name, see S1 Table). After the chimeric gene was inserted at a benign location in the nuclear genome of the wild-type strain and the native *Atp9-5* locus was deleted (see S1 Table), the resultant oligomycin-sensitive strain, ^*7*^*7[*^*5*^*7]*, was mutagenized with UV light and screened for growth in the presence of the antibiotic. This effort led to the acquisition of a fungal mutant that grew very well on oligomycin plates (strain ^*7*^*7[*^*5*^*7*_*OR*_*]* in Fig 2B; see also S3 Table) as the result of an *O*_*R*_ mutation in the chimeric ^*5*^*7* gene that caused a F135Y substitution in the ATP9-7 *c*-subunit.

Finally, the strains ^*7*^*7[*^*5*^*5]*^*5*^*5*_*OR*_ and ^*7*^*7[*^*7*^*5]*^*5*^*5*_*OR*_ were constructed by introducing a ^*5*^*5* or a ^*7*^*5* transgene into the ^*7*^*7*^*5*^*5*_*OR*_ mutant (S1 Table). Analysis of these strains showed that the F124S substitution exhibited partial dominance *versus* the wild type allele, and conferred growth on oligomycin plates, if transcription of the latter was regulated by *Atp9-7* flanking sequences but not if the regulatory elements derived from *Atp9-5* (Fig 2B, S3 Table). For a strain to be oligomycin-resistant, the *c*-ring of the F-ATP synthase in apical cells must be composed 100% of the mutant protein because the incorporation of as little as one wild type *c*-subunit monomer would render the enzyme sensitive to the antibiotic when facing the *a*-subunit. The oligomycin-resistant phenotype observed for strain ^*7*^*7[*^*7*^*5]*^*5*^*5*_*OR*_ indicates that the level of wild type *c*-subunit in the proliferating cells at the apex is insignificant when expression of the gene is regulated by the 5’ and 3’ flanking sequences from *Atp9-7*.

### The ATP9-5 and ATP9-7 proteins have antagonist effects on the longevity of *P*. *anserina*

**Fig 3:**
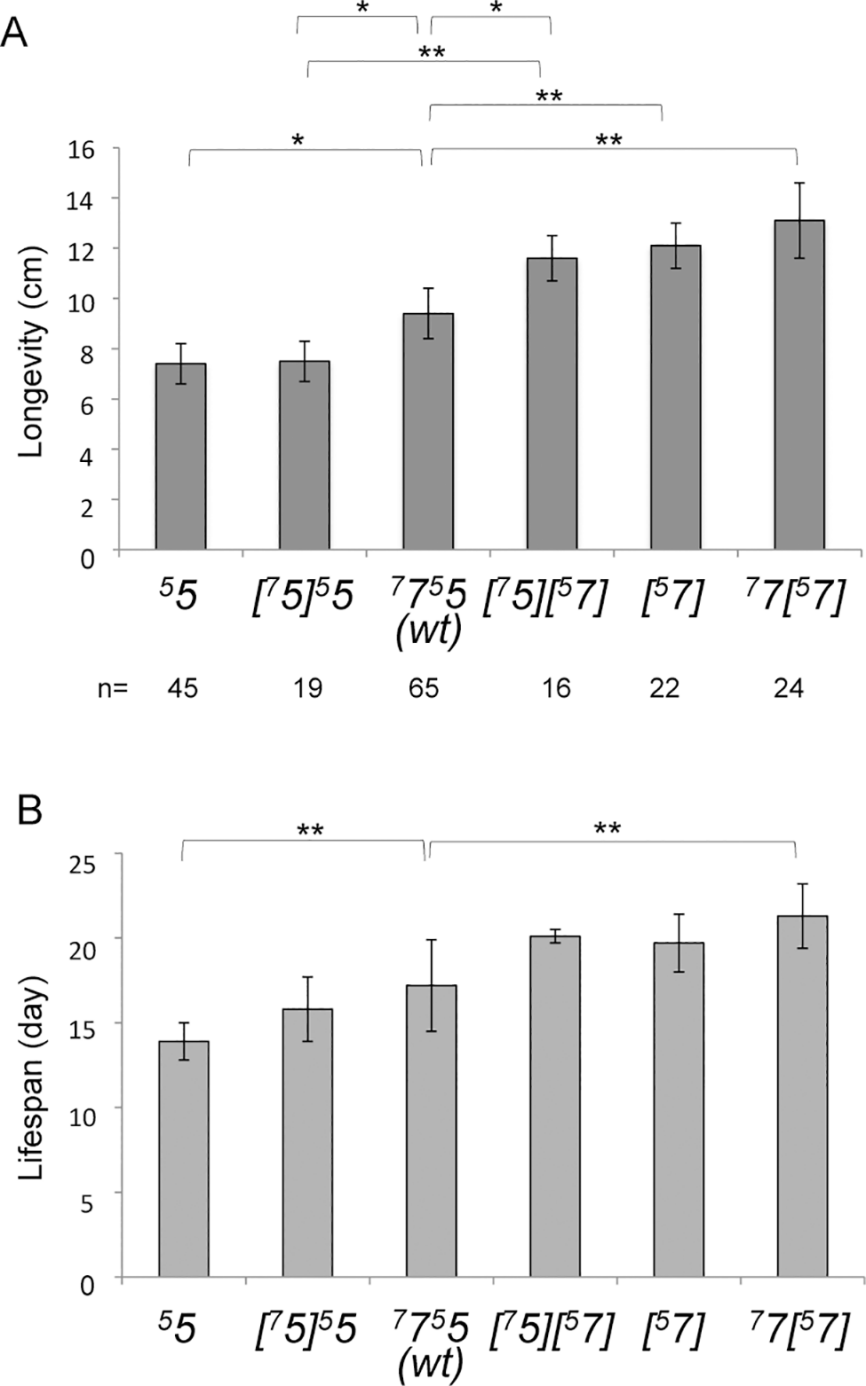
The ATP9-5 and ATP9-7 proteins have antagonist effects on the longevity of *P*. *anserina*. This figure reports the life span values for strains in which the expression of *Atp9-5* and/or *Atp9-7* is regulated differently than in wild type, as evaluated by the linear length (in cm) the mycelium reaches before dying (Panel A), or by the estimation of the half-life (in days), which is the number of days by which 50% of the cultures were still alive (panel B) (see S3 Table for details). The reported values and standard deviations were established by testing at least 32 cultures for each genotype obtained from several independent crosses (S3 Table). Statistically significant changes in longevity between strains, according to t-student and log rank tests are indicated by the bars and stars (* corresponds to a *P*-value <95%, ** to a *P*-value <0.01). The short-hand nomenclature of the analyzed strains is explained in the legend of Fig 2 (see S1 Table for complete genotypes).

We previously constructed a strain of *P*. *anserina* in which *Atp9-5* expression is controlled by *Atp9-7* regulatory sequences and *vice versa* for *Atp9-7* (strain *[*^*7*^*5][*^*5*^*7]*), and showed that it completes the full life cycle [7]. We interpreted this finding to indicate that the two *c*-subunit isoforms are equivalent functionally. However, having now examined the vegetative growth stage in greater detail, a functional difference between isoforms encoded by *Atp9-5* and *Atp9-7* was made apparent. Indeed, swapping the regulatory sequences of *Atp9-5* and *Atp9-7* (strain *[*^*7*^*5][*^*5*^*7]*) leads to a significant increase in longevity with respect to the wild type (123%, p<2x10^-5^, Fig 3A and S3 Table), as evaluated by the linear length the mycelium reaches before dying. To pursue further the idea that selective expression of *Atp9-7* at the apex causes an increase of longevity, the life span was measured for additional strains in which the expression of *Atp9-5* and/or *Atp9-7* is regulated differently than in wild type. Compared to wild type, the life span was reduced significantly for strain ^*5*^*5* (79%, p<5x10^-5^, Fig 3 and S3 Table), which is completely deleted for *Atp9-7* and this phenotype persisted in strain *[*^*7*^*5]*^*5*^*5*, which harbors a second copy of the *Atp9-5* coding sequence that is flanked by *Atp9-7* regulatory sequences (S1 Table). Instead, for the case in which the only source of the *c*-subunit is *Atp9-7*, fungal longevity was increased (129%, p<3x10^-6^, Fig 3 and S3 Table) as long the strain carries a copy of the gene that is regulated by *Atp9-5* sequences (strains *[*^*5*^*7]* and ^*7*^*7[*^*5*^*7]*). Cumulatively, these results show that the exclusive production of *c*-subunit encoded by *Atp9-7* correlates with an increased fungal life span while longevity is compromised in strains for which production of the *c*-subunit is limited to the *Atp9-5* coding sequence. Estimation of the life span by the number of days by which 50% of the cultures were still alive (half-live) further supported this conclusion (Fig 3B, S3 Table).

### The ATP9-5 and ATP9-7 proteins confer different properties to the F-ATP synthase in *P*. *anserina*

The results of the longevity experiments were interesting in light of previous work that showed the life span of *P*. *anserina* is sensitive to factors that impact mitochondrial oxidative phosphorylation: fungal longevity was increased in mutants carrying a genetic defect linked to one of the respiratory complexes [19–24] (see Discussion). We hypothesized that some aspect of the F-ATP synthase related to fungal bioenergetics and life span might be modified by the incorporation of different *c*-subunit isoforms in its structure. *P*. *anserina* strains *[*^*5*^*7]* and ^*5*^*5* (described above, see S1 Table) were ideally suited for experiments to investigate this idea, first because each strain is genetically pure for only one of the *c*-subunit isoforms. Second, the regulatory sequences from *Atp9-5* control the gene transcription for both types of *c*-subunits and this guarantees uniformity between strains with respect to the amount of *c*-subunit mRNA that is produced in the apical cells of the mycelial cultures (S2 Fig).

**Fig 4:**
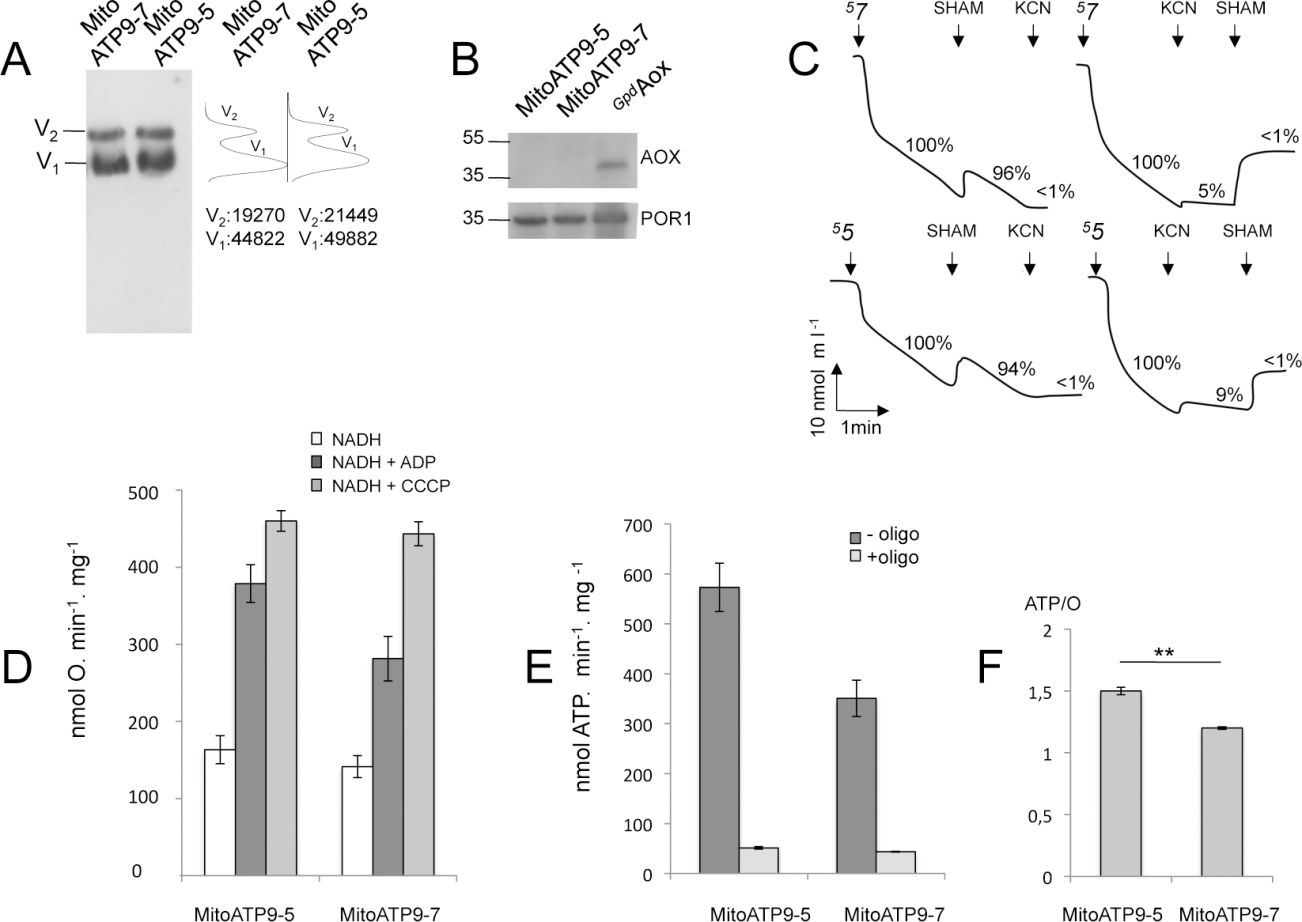
The ATP9-5 and ATP9-7 proteins confer different properties to the F-ATP synthase in *P*. *anserina*. All of these experiments were performed using mitochondria isolated from the apical cells of strains *[*^*5*^*7]* and ^*5*^*5*, which express either *Atp9-5* or *Atp9-7* both from the regulatory sequences of *Atp9-*5, except in panel (C) where protoplasts prepared from these strains were used. For simplicity, the mitochondrial samples are referred to as MitoATP9-5 and MitoATP9-7, whereas the protoplasts are named ProtoATP9-5 and ProtoATP9-7, respectively, to denote which *c*-subunit isomer was produced in the cells of origin. (A) BN-PAGE analysis of ATP synthase. *On the left*: Mitochondrial proteins were extracted with 2% digitonin, separated by BN-PAGE (50 μg per lane) and transferred to nitrocellulose membranes for Western blotting with antibodies against the yeast α-F_1_ protein. In the conditions used, ATP synthase was detected as dimeric (V_2_) and monomeric (V_1_) units. *On the right*: Quantification of the immunological signals (see Materials and Methods). The values correspond to the surface areas below the V_2_ and V_1_ peaks. The two protein samples loaded on the BN-gel contained similar amounts of porin, as shown in panel B. A second, independent, BN-PAGE analysis is shown in S4 Fig (B) Steady state levels of porin and AOX. 50 μg of proteins from MitoATP9-5 and MitoATP9-5 were separated *via* SDS-PAGE, transferred to a nitrocellulose membrane and probed with antibodies against porin and AOX. ^*Gpd*^*Aox* mitochondria were isolated from a strain that constitutively expresses AOX [35]. C. Sensitivity of oxygen uptake to SHAM. 2x10^8^ protoplasts prepared from strains *[*^*5*^*7]* (ProtoATP9-7) and ^*5*^*5* (ProtoATP9-5) were pre-incubated for 30 min in the respiration buffer and their oxygen consumption activities were then measured using a Clark electrode. SHAM and KCN inhibitors were used at 1mM. The respiration value in the absence of inhibitor is set-up as 100%. The residual respirations after adding the adding the inhibitors are indicated. (D) Oxygen uptake by MitoATP9-5 and MitoATP9-7. Measurements with a Clark electrode were made with mitochondria at a protein concentration of 0.15 mg/ml, in the presence of NADH alone (basal respiration), and after subsequent additions of 150 μM ADP (state 3) or 4 μM CCCP (uncoupled respiration). (E) ATP synthesis. This activity was evaluated in the conditions used to measure state 3 respiration except that 1 mM instead of 150 μM ADP was added to the mitochondrial samples. (F) The histogram reports the ATP/O values calculated for MitoATP9-5 and MitoATP9-7, which is the number of ATP molecules produced per pair of electrons transferred to oxygen (see S4 Table for details; ** is for p<0.01%). The data reported in panels C, D and E are the mean values ± standard deviation obtained in at least three independent experiments (see S4 Table for details).

Mitochondria were isolated from the apical cells of strains *[*^*5*^*7]* and ^*5*^*5*. For simplicity, the samples are hereafter referred to as MitoATP9-7 and MitoATP9-5, respectively to denote which *c*-subunit isomer was produced in the cells of origin. BN-PAGE analyses revealed that the F-ATP synthase was in similar, if not identical, amounts in MitoATP9-5 and MitoATP9-7 samples that contained the same amount of porin (Fig 4A and 4B; and S4 Fig where quantification of ATP synthase from two independent experiments is provided). Oxygen consumption and ATP synthesis rates were measured using NADH as a respiratory substrate. It is to be noted that external NADH cannot be oxidized by complex I because the catalytic site of this complex is located in the mitochondrial matrix [25]. NADH can nevertheless deliver electrons to ubiquinone (Q) *via* two monomeric NADH dehydrogenases (NDE1 and NDE2) located on the outer side of the inner membrane and these electron transfers are not coupled to the translocation of protons across the mitochondrial inner membrane [19]. Thus, using external NADH, the proton gradient for ATP synthesis is generated by electron transfer from reduced ubiquinone (QH_2_) to oxygen, through respiratory complexes III and IV, which is coupled to the pumping of protons out of the mitochondrial matrix. A critical factor for interpreting these experiments was that the respiratory data could be used quantitatively provided there was not another pathway for oxygen consumption in the mitochondria. On this point, *P*. *anserina* contains a gene (*Aox*) encoding an alternative oxidase (AOX) that bypasses complexes III and IV and transfers electrons directly from ubiquinol to oxygen without generating a proton gradient. In other words, AOX consumes oxygen in a reaction that is not coupled to ATP synthesis and, if present in MitoATP9-5 or MitoATP9-7 mitochondria, would complicate calculations of oxidative phosphorylation parameters. Fortunately this gene is expressed in the fungus only under peculiar conditions, for instance when the external medium is poisoned with chemicals (like antimycin, myxothiazol, cyanide) that inhibit complexes III or IV and in strains with loss-of-function mutations in complex III or complex IV [19–24]. In accord with our expectations, there was hardly any evidence of *Aox* transcripts detected by qRT-PCR with mycelial RNA samples from four different fungal strains, including those (*[*^*5*^*7]* and ^*5*^*5*) used to isolate mitochondria for the studies described in this section (S2 Fig, bottom panel). In fact, the *Aox* mRNA level was even too low to be quantified relative to the same control transcript (*Gpd*) used to quantify *Atp9-5* and *Atp9-7* expression (S2 Fig, top and middle panels). For this reason the *Aox* transcript levels are reported relative to those from a poorly expressed control gene (*Pdf2*) and shown on a different scale in the bottom panel of the figure. Consistent with these transcript analyses, we failed to detect by Western blot the AOX protein in the MitoATP9-5 and MitoATP9-7 samples (Fig 4B). Finally, respiration was in both mitochondria only minutely (5%) inhibited by SHAM, a specific inhibitor of AOX (Fig 4C). As such, we are confident that oxidative phosphorylation was the only significant pathway available for oxygen consumption in the MitoATP9-5 and MitoATP9-7 samples.

Oxygen consumption (Fig 4D) and ATP synthesis rates (Fig 4E) in the presence ADP (state-3) were significantly higher in MitoATP9-5 *versus* MitoATP9-7 (see also S4 Table). However, the similar rates in oxygen consumption that were measured for both samples in the presence of an uncoupling agent (CCCP) indicated that the maximal respiratory capacity and potential to generate a proton motive force were actually quite comparable (Fig 4D, S4 Table). In contrast, the capacity of the F-ATP synthase to use the proton motive force for ATP synthesis differed significantly; the yield in ATP *per* pair of electrons transferred to oxygen was higher in MitoATP9-5 with respect to MitoATP9-7 (1.51 *versus* 1.25, Fig 4F). Most importantly, the differences were reproducibly observed in three independent experiments (S4 Table). With equivalent amounts of F-ATP synthase, the *c-*subunit was the only factor relevant to energy coupling that was different between the MitoATP9-5 and MitoATP9-7 samples. The most straightforward interpretation of these experiments is that in *P*. *anserina* different isoforms of the *c*-subunit are incorporated in the F-ATP synthase to regulate the ATP/O ratio and control how energy is utilized by cells.

## Discussion

A previous paper reported that the *c*-subunit of the mitochondrial F-ATP synthase is encoded by two paralogous genes, *Atp9-5* and *Atp9-7*, in the nuclear genome of the filamentous fungus *P*. *anserina* [7]. The results from early work to characterize isogene expression showed that both genetic loci were active throughout the fungal life-cycle [7]. Also, *Atp9-7* proved to be transcribed preferentially during spore maturation while *Atp9-5* expression was clearly dominant in germinating spores. We have since completed a detailed transcriptional analysis in vegetative mycelium with respect to both the levels of *Atp9-5* and *Atp9-7* mRNAs (Fig 1) and properties of the 5’ and 3’ sequences that flank the *Atp9* coding sequences (Fig 2). The reason for the profound difference in the level of *Atp9-5* transcripts between whole mycelium samples on the first and last days (w-1d, w-5d) was not immediately clear. Though if *Atp9-5* expression was limited to only the proliferating cells, the data trend was in accord with the diminishing contribution of these to the total fungal mass as the mycelium expanded radially. Since gross dissection of peripheral region from whole mycelium (Fig 1, p-5d) does not provide a pure sample of only the actively proliferating cells, an accurate description of the expression pattern necessitated the preparation of protoplasts originating from apical cells. Such samples faithfully reported the properties exclusive to apical cells. Combining the results of these samples (a-2d, a-5d) with those from RNAs that were isolated from the stationary phase mycelium removed from the center of the thallus (c-5d) reveals clearly that the expression of *c*-subunit isogenes in *P*. *anserina* tracks with the mitotic status of the cells; the non-proliferating cells, which comprise most of the vegetative apparatus in developed mycelium, accumulated a steady state level of *Atp9-7* transcripts and little, if any, from the other gene, while *Atp9-5* was preferentially and very highly expressed in the growth zone where the apical cells of filaments are located.

Chimeric genes in which the coding sequence for a reporter protein is flanked with the upstream and downstream regions from either the *Atp9-5* or *Atp9-7* provided insight to how the isogenes are regulated *in vivo*. In short, only the sequences from *Atp9-5* permitted expression of the chimeric genes at a high enough level in order to initiate and support growth on nourseothricin (Fig 2A) or oligomycin (Fig 2B). These findings indicated that the near absence of *Atp9-7* transcripts from apical cells is solely a function of the flanking sequences, which do not respond to a transcriptional activation signal (or signals) in apical cells, and has nothing to do with the coding region of the gene. We wish to point out here that the impetus to mutagenize *P*. *anserina*, both the wild type (^*7*^*7*^*5*^*5*) and the strain that was optimized to enable high production of the *c*-subunit encoded by *Atp9-7* in the proliferating cells at the apex (^*7*^*7[*^*5*^*7]*), was to isolate oligomycin-resistant alleles of both *c*-subunit isomers for construction of the chimeric gene reporters.

In fact, the mutant alleles that encode the *c*-subunit with either the F124S substitution (in ATP9-5) or F135Y substitution (in ATP9-7) are interesting in their own right and relevant to the broad field of ATP synthase bioenergetics. The efficacy of oligomycin resistance, which we observed for single substitution at either F124 or F135, was not surprising because both phenylalanine residues are identically conserved in the *c*-subunit from *Saccharomyces cerevisiae* (numbered F55 and F64 in the yeast protein) and map to the oligomycin binding site in the ATP synthase of this organism ([15], see S5 Fig). Moreover, published work had already identified an oligomycin-resistance conferring mutation affecting F64 in the yeast *c-*subunit [16]. Hence, we are not the first to show that a single mutation of the amino acid that occupies the position of F135 in the *c*-subunit of *P*. *anserina* is sufficient to prevent oligomycin from interfering with proton translocation in the ATP synthase.

On a conceptual basis, the expression pattern of *Atp9-5* and *Atp9-7* in mycelium was similar to that observed during early development [7]; the two isogenes were differentially expressed in individual cell populations, as a function of differences in the regions that flank the protein coding sequences, and the amount of mRNA for the *c*-subunit was significantly higher in active *versus* resting cells. This regulatory strategy has important implications for aerobic energy metabolism because the cellular content of F-ATP synthase can be controlled by how much *c*-subunit is available. Together these results indicate that the transcriptional regulation of *Atp9-5* and *Atp9-7* is used to modulate ATP synthase production during the life cycle of *P*. *anserina*: when large amounts of ATP synthase need to be produced, *i*.*e*. during spore germination and vegetative growth, the *c*-subunit is mainly, if not exclusively, expressed by *Atp9-5*, whereas *Atp9-7* becomes the main source of *c*-subunit in sedentary cells and maturing spores that require a lesser amount of newly-synthesized F-ATP synthase.

While it makes intuitive sense that ATP synthase production needs to be modulated along the life cycle of *P*. *anserina*, it was not at all obvious why as part of this strategy two different *c*-subunit gene isoforms are used. Indeed, one single gene controlled by a nutrient sensing system could *a priori* be sufficient. For example in humans and other mammals, there is a single species of the *c*-subunit that is produced from three different genetic loci, in which unique characteristics of the flanking sequences allow the modulation of ATP synthase levels in a cell- and tissue-specific manner [26]. The results from our experiments with mitochondria isolated from strains that produced equivalent amounts of one or the other *c-*subunit isoform have provided initial evidence of a two-pronged regulatory mechanism that is based not only on the availability of *c*-subunit but also on characteristics of the protein itself. Despite the similarities in ATP synthase content and rates of oxygen consumption in the presence of CCCP, the ATP/O ratio was measured to be 1.51 in MitoATP9-5 and 1.25 in MitoATP9-7 (Fig 4, S4 Table). The implication of this finding is that the individual *c*-subunit isomers might confer different functional properties to the ATP synthase. Indeed, the two mitochondrial samples show almost the same passive permeability to protons as evidenced by their similar state 4 respiration rates. One way the ATP9-7 and ATP9-5 proteins could modify the functional properties of the ATP synthase is through modification of the seal between the central stalk of F_1_ and the *c*-ring caused by structural differences in the two proteins. One could also speculate that the lower RCR value in MitoATP9-5 compared to MitoATP9-5 is due to lower maximal turnover of ATP synthase under load (in contrast to the uncoupled state), which could be an alternative functional difference between the two forms of the enzyme. Alternatively, *c*-rings assembled from the *c*-subunit encoded by *Atp9-5* might contain less monomer than those made from the *c*-subunit encoded by *Atp9-7*. As a result, fewer protons would be needed to make one ATP with the former than with the latter. Recent studies have shown that the *c*-ring stoichiometry is quite variable among species (from 8 to 15) due to structural specificities in the N-terminal α-helix of *c*-subunit [27–32]. Although it is impossible to predict the *c*-ring stoichiometry from the primary sequences of the proteins encoded by *Atp9-5* and *Atp9-7*, it is interesting to note that amino-acid differences exist between them in positions presumed to be critical for the packing of *c*-subunits (S5 Fig). It will be interesting in the future to determine the efficiency of translation of ATP9-5 and ATP9-7 and the turnover of the two forms of ATP-synthase, which could reveal an additional level of regulation that could explain why two structurally different *c*-subunit isomers are used in *P*. *anserina*. While much remains to be learned, one thing is clear: by invoking different *c*-subunit isoforms *P*. *anserina* demonstrates a degree of sophistication for regulating aerobic energy metabolism that has not been described before.

There is a large body of observations in the literature indicating there is a link between longevity and mitochondrial function in many organisms including *P*. *anserina* (for review, see [33]). For instance, mutants of this fungus severely defective in complex I, III or IV, display a huge increase in longevity [19–21,34]. It has been argued that the induction of AOX that occurs in these mutants was possibly involved by some mechanism in the observed changes in longevity. However, while AOX becomes essential for cellular viability when electrons can no longer be transferred to oxygen by complexes III and IV, the deletion of the AOX gene is tolerated well by complex I mutants and these remained long-lived [19]. Interestingly, overexpressing NDI1, an alternative mitochondrial NADH dehydrogenase, and AOX partially reversed the longevity phenotypes of complexes I, III and IV mutants [19,35,36], indicating that the rate of electron flow is a key factor that influences longevity. According to the ‘mitochondrial free radical theory of aging’ [37], increasing this flow possibly enhances the production of reactive oxygen species (ROS) that can damage any type of biomolecules, owing to a higher diversion of electrons from their normal pathway to oxygen. While supported by many data, this theory was however challenged by controversial results [38]. For instance, no increase in carbonylation of mitochondrial proteins was observed during aging of *P*. *anserina* [39], and eliminating the gene of the mitochondrial localized ROS detoxifying protein PaSod3 does not affect life span [40]. Furthermore, the long-lived complex I mutants of *P*. *anserina* do not show any change in production and/or scavenging of free radical species [19]. These observations point to the rate of production of ATP, the end product of respiration, as a possible life span modulator. The results of this study support this hypothesis. Indeed, reducing the rate of ATP production in apical cells by expressing there *Atp9-7* instead of *Atp9-5* leads to a longer life span while the mitochondrial electron transfer capacity remains unchanged.

## Materials and Methods

### Strains

All the strains are derived from the *S* wild type strain of *Podospora anserina* that harbors two different genes encoding the *c-*subunit of ATP synthase: *Atp9-5* (Pa_5_9140) and *Atp9-7* (Pa_7_20). The short-hand nomenclature of the strains describes what allele is actively transcribed (5 and 7 in regular point size) and the origin of the 5’ and 3’ flanking sequences that control expression of the alleles (5 or 7 in superscript just before the allele):

^*5*^*5* ^*7*^*7* is for the wild type strain. The wild type (^*5*^*5* and ^*7*^*7)* and the inactivated (*Δ5* and *Δ7* that do not appear in the name of the strain) *Atp9* alleles have been combined with various *Atp9* ectopic transgenes expressed under the control of switched *cis*-regulatory sequences (^*5*^*7*) and (^*7*^*5*) by successive genetic crosses [7]. The previous nomenclature of the strains was modified and adapted for coherence with this manuscript: ^*7*^*7[*^*5*^*7]* (instead of *Δ5 7*^*5*^), *[*^*7*^*5][*^*5*^*7]* (*Δ5 Δ7 5*^*7*^ *7*^*5*^), *[*^*7*^*5]*^*5*^*5* (*Δ7 5*^*7*^), *[*^*5*^*7]* (*Δ7 Δ5 7*^*5*^) and ^*5*^*5* (*Δ7*). The selection of strains with oligomycin resistant *Atp9* alleles *(*^*5*^*5*_*OR*_ *and [*^*5*^*7*_*OR*_*]*), the construction of strains with new transgenes (^*5*^*5*, ^*5*^*nat*, ^*7*^*nat)* and their combination by genetic cross to obtain the various strains used in this work are detailed in Supporting Information (S1 Methods). Complete genotypes and nomenclature of all strains are detailed in S1 Table.

### Vegetative growth and longevity measurements

Vegetative cultures were initiated from small pieces of mycelium freshly obtained from germinated spores. They were grown at 27°C in Petri dishes containing solid M2 minimal media supplemented, when necessary, with antibiotics (http://podospora.igmors.u-psud.fr/ and Text SI). When mycelium had to be collected (*e*.*g*. for protoplast preparation or RNA extraction) it was grown on plates overlaid with cellophane.

For longevity measurements at least 32 independent cultures for each tested genotype (see S1 Methods, S3 Table) were grown on solid M2 medium in 30cm-race tubes. Longevities were determined in centimeters of linear growth from the culture initiation until the apical front stops growing or by the estimation of the half-life (in days), which is the number of days by which 50% of the cultures were still alive.

### Protoplasts preparation and mitochondria extraction

For each strain, 20 liquid cultures were initiated with fragmented mycelium as described (see S1 Methods for details). Briefly, the grown mycelium was drained and cell walls were digested for 3 hours with 40 mg/ml Glucanex (Novozyms), after which the protoplasts were collected by filtration and centrifugations.

Mitochondria were released from 10^9^ protoplasts by osmotic shock in 0.33 M saccharose, 1 mM EGTA, 0.2% BSA pH 6.8 at 4°C. Mitochondria were isolated by differential centrifugation 4°C in the same buffer, washed, and suspended at 10 mg/ml in the same buffer. The procedure yielded ~1.5 mg mitochondrial protein per ~200 g starting material. Protein concentrations were determined using the BIORAD protein assay.

### Oxygen consumption and ATP synthase activities

Oxygen consumption was measured with a Clark-type O2 electrode (Hansatech) at 28°C in a 1 ml-oxytherm thermostated chamber that contained 0.65 M mannitol, 0.36 mM EGTA, 10 mM Tris/maleate, 5 mM Tris/Phosphate pH 6.8 and 0.3% BSA. Freshly prepared mitochondria and NADH were added to 0.15 mg/ml and 4 mM, respectively. State 3 respiration was initiated with ADP (150 μM) and the reaction was followed until the system converted back to the resting rate of respiration (state 4), which indicated that all of the ADP had been phosphorylated. The maximal (uncoupled) rate of respiration was measured with carbonyl cyanide *m*-chlorophenyl hydrazone **(**CCCP 5μM) included in the reaction mix. The sensitivity of respiration to SHAM was measured with protoplasts prepared from apical cells (see S1 Methods) before or adding KCN. KCN and SHAM were each used at a concentration of 1mM. For measurement of ATP synthesis rates, the chamber buffer contained excess ADP (1mM). Samples (50 ul) were removed at 15 second intervals and added to a solution of 2.3% perchloric acid and 8.3 mM EDTA to stop the reaction. Acid-quenched samples were brought to pH 6.5 with 0.3 M MOPS, 2 N KOH and the amount of ATP was determined using a luciferin/luciferase assay (ATPlite 1step, PerkinElmer; Turner Scientific (Reporter) bioluminometer).

### Immuno-detection

Published methods [41,42] were used for electrophoretic separation of mitochondrial proteins on 12% SDS-polyacrylamide or 5–10% Blue Native (BN) gels. For BN-PAGE analysis, mitochondria were solubilized with 2% digitonin, and after the electro-transfer of the proteins onto nitrocellulose membrane ATP synthase was detected using polyclonal antibodies against yeast α-F_1_ protein (a gift from J. Velours) at a 1:10,000 dilution. The immunological signals obtained on a X-ray film were scanned as 16-bit tiff images and quantification using ImageJ software [43]. Antibodies against porin and AOX (provided by H.D. Osiewacz and T. Elthon) were used after 1:5000 and 1:100 dilutions, respectively.

### Real-time qRT-PCR

Mycelium cultures were initiated from single pieces of mycelium on solid M2 medium overlaid with a cellophane sheet and grown at 27°C for 1, 2 or 5 days. Depending on the experiment, the entire mycelium, or only part of it, was collected with a scalpel blade, added to a screw-capped tube, and disrupted with glass beads. Alternatively, 5x10^7^-10^8^ protoplasts acquired from 2 or 5 day-liquid cultures were used. Mycelium and protoplasts were frozen in liquid nitrogen then ground in the Qiagen RLT buffer in a FasPrep apparatus (speed 6 for 45sec; MP Biomedical). Total RNA was extracted using the RNAeasy plant kit (Qiagen). The number of thalli collected was adapted according to the time of culture (24 hours-5 days) in order to obtain 25–50 μg RNA. Quantification of transcripts was conducted in a two-step procedure. Reverse transcription of 2 μg total RNA was performed using the SuperScript II (Invitrogen) kit and primed with oligo(dT)_15_. cDNA levels were then quantified by real time PCR in the Light Cycler 480 system using couples of primers described in S5 Table and reaction kits containing SYBR Green I (Roche). Data were analyzed using the ‘second derivative maximum’ method for quantification. Amplification efficiency was determined for each couple of primers, based on standard curves established using serial dilutions of one of the cDNA samples. The abundance of *Atp9-5* and *Atp9-7* transcripts in at least three independent RNA extractions was estimated relative to that of the constitutively expressed *Gpd* gene *(*Pa_3_5410). Results were expressed as mean values relative to the abundance of *Atp9-7* in a one day-old culture of the wild type (w-1d, see Fig 1).

It is to be noted that during protoplast preparation living cells are subject to a long, possibly stressful, period (3 hours in the presence of glucanex; see S1 Methods for details) that might affect gene expression. We therefore tested the influence of the procedure by repeating the transcript analyses on w-5d mycelium exposed to these conditions before extracting RNA. The relative abundance of *Atp9-5* and *Atp9-7* transcripts was essentially unchanged compared to that measured without exposure of the mycelium to these conditions (S3 Fig), which precludes any procedural bias.

## Supporting Information

S1 Methods (DOCX)

S1 Table Genotypes of the strains. For each strain, the allelic state of *Atp9-7* and *Atp9-5* and the presence of ectopic transgene(s) are presented. Superscripts denote the origin of the 5’ and 3’ *cis-*regulatory sequences (~500–600 nucleotides upstream the ATG for 5’ and ~600–800 nucleotides downstream the stop codon for 3’). Inactivated alleles (Δ) and transgenes are linked to an antibiotic-resistance cassette containing either *hph* (hygromycin-resistance), *bl*e (phleomycin resistance) or *nat1 (*nourseothricin-resistance) genes. *OR* is for oligomycin-resistance conferred by the F124S or F135Y mutation in ATP9-5 and ATP9-7, respectively.
(DOCX)

S2 Table Phenotypes of the *nat* transgenic strains. ^a^ Full genotypes of strains are given in S4 Table. ^b^ Germination efficiency in the absence of nourseothricin (nourseo) is expressed as the percentage of spores that germinated within a period of 7 days on G medium compared to the wild type strain. The germination efficiencies measured in the presence nourseothricin are for each strain expressed relative to those obtained without the drug.
(DOCX)

S3 Table Phenotypes of the *Atp9* mutant strains. ^a^ The full genotype of the strains is given in S2 Table. ^b^ Sporulation data were reported previously [7]: The efficiency of spore production is estimated counting the number of spores ejected in homozygous crosses. +++, ++, + and–correspond to 85–100%, 60–80%, 20–40% and less than 1% respectively. ^c^ Germination efficiency is expressed as the percentage of spores that germinated within a period of 7 days on G medium compared to the wild type strain. ^d^ Density of the mycelium (weight/area) was estimated on mycelium grown for 3 days. ^e^ Oligomycin resistance corresponds for each strain to the percentage of growth in presence of oligomycin compared to the growth without the drug (5–15 cultures). ^f^ Longevities are mean values estimated from at least 32 cultures for each analyzed strain, expressed in centimeters (cm) of growth reached when the strain died and the number of days (half-life or median life span) by which 50% of the cultures were still alive. nd: not determined, n: number of independent cultures.
(DOCX)

S4 Table Oxidative phosphorylation in MitoATP9-5 and MitoATP9-7. MitoATP9-5 and MitoATP9-5 extracts were prepared from protoplasts of strains ^*5*^*5* and *[*^*5*^*7]*, respectively. Three independent experiments for each strain were performed. Oxygen consumption was determined using NADH as a respiratory substrate, alone (state) 4 or in the presence of ADP (state 3) and in the presence of CCCP (uncoupled respiration). The respiratory control ratio (RCR) corresponds to the ratio between state 3 and state 4 respiration rates. ATP/O is the number of ATP molecules formed per oxygen atom reduced. Mean data are given with standard deviation.
(DOCX)

S5 Table List of primers. Gene nomenclature is according to the *Podospora anserina* Genome Project (http://podospora.igmors.u-psud.fr/). Fw and Rev correspond to the couples of primers used for qRT-PCR experiments. In each couple of primers one (*) overlaps an exon-exon junction to minimize non specific amplification.
(DOCX)

S1 Fig Adding an HA tag at the C-ter of ATP9-5 compromises its ability to complement Δatp9 yeast. As we have shown previously [11], the *Atp9-5* gene fused to a mitochondrial targeting sequence restores the capacity of a yeast strain lacking the mitochondrial *ATP9* gene (**Δ**atp9) to grow on respiratory substrate (glycerol). (A) Growth curves on glycerol showing that with an HA tag at its C-ter ATP9-5 can no longer restores mitochondrial function in **Δ**atp9 yeast. (B) Western blots of total protein extracts with antibodies against porin, HA epitope, and yeast Atp9p (yATP9) showing ATP9-5.HA accumulation in Datp9 yeast transformed with the tagged gene.
(TIF)

S2 Fig Transcriptional activity of *Atp9-5*, *Atp9-7* and *Aox* in strains ^*7*^*7*^*5*^*5* (*wt*), ^*5*^*5*, *[*^*5*^*7]* and *[*^*7*^*5][*^*5*^*7]*. RNA extracts were prepared from whole mycelium cultured on solid media for 2 days (w-2d), or 5 days (w-5d) (see Fig 1). The levels of mRNA transcripts from *Atp9-5*, *Atp9-7* and *AO*X were determined by real-time quantitative reverse transcription PCR using, as indicated *Gpd* or *Pdf2*, as a reference gene.
(DOCX)

S3 Fig Relative abundance of *Atp9-5 and Atp9-7* transcript in mycelium exposed or not to conditions used for preparing protoplasts from apical cells. These measurements were made using 5-day-old mycelium (w-5d) of the wild type strain of *P*. *anserina* grown on solid plates. On the left (w-5d) are the results obtained with RNA extracts prepared from non-treated mycelium; on the right (w-5d gluc) are those obtained when RNAs were extracted from mycelium treated 3 hours in the presence of glucanex (conditions used to prepare protoplasts a-2d and a-5d in Fig 1). The constitutively expressed *Gpd* gene was used as a reference gene.
(DOCX)

S4 Fig Quantification of ATP synthase complexes in Mito-ATP9-5 and MitoATP9-7. (A) BN-PAGE analysis of ATP synthase. *On the left*: Mito-ATP9-5 and MitoATP9-7 samples were extracted with 1% n dodecyl β D-maltoside (Sigma D4641), separated by BN-PAGE (50 μg per lane) and transferred to nitrocellulose membranes for Western blotting with antibodies against the yeast α-F_1_ protein. In the conditions used, ATP synthase was detected as dimeric (V_2_) and monomeric (V_1_) units. Below the blot are the results of the quantification of the immunological signals corresponding to V_1_ and V_2_ normalized to porin (see Materials and Methods). (B) 50 μg of proteins from MitoATP9-5 and MitoATP9-7 were separated *via* SDS-PAGE, transferred to a nitrocellulose membrane and probed with antibodies against porin. Below the blot are the results of the quantification of the immunological signals corresponding to porin (see Materials and Methods). (C): Mean values of the contents in V_2_ and V_1_ normalized to porin in the BN-PAGE experiments shown in Fig 4A and the one shown in panel A of this figure.
(DOCX)

S5 Fig Alignement of the proteins encoded by the *Atp9-7* and *Atp9-5* genes of *Podospora anserina* with *c*-subunits of known stoichiometry from various origins. The shown alignment was established with ClustalW. The aligned protein sequences with a mitochondrial (mt) origin are from *Homo sapiens* (HOMSA), *Bos Taurus* (BOSTA), *Drosophila melanogaster* (DROME), *Saccharomyces cerevisiae* (SACCE), and *P*. *anserina* (PODATP9-5, PODATP9-7); those with a bacterial origin are from *Bacillus pseudofirmus* (BACPSOF4 and BACPSSP3), *Ilyobacter tartaricus* (ILYTA), *Clostridium paradoxum* (CLOPA), *Synechoccus elongatu*s (SYNEL), *Caldalkalibacillus thermarum* (CALTH), *Spirula platensis* (SPIPL); the last sequence, from *Spinacea oleracea* (SPIOL), has a chloroplastic (chl) origin. The black arrows point to the Phenylalanine residues mutated in the oligomycin-resistant strains of *P*. *anserina* described in this study (F124S in ATP9-5; F135S in ATP9-7). The asterisk points to the essential Glutamate residue that is protonated/deprotonated during catalysis. Positions in the *c*-subunit, presumed to be important for the *c*-ring stoichiometry [27,44], are indicated by grey arrows.
(DOCX)
