# Supplementary material for: Regulation of Aerobic Energy Metabolism in Podospora anserina by Two Paralogous Genes Encoding Structurally Different c-Subunits of ATP Synthase
Source: PLoS Genet. 2016 Jul 21;12(7):e1006161. doi: 10.1371/journal.pgen.1006161 (PMC4956034; doi:10.1371/journal.pgen.1006161)
Supplement: S1 Methods — (DOCX) [file pgen.1006161.s001.docx]

**SI Methods**

**Chromosomal integration of transgenes**. The transgenes were co-integrated in chromosomal DNA with a phleomycin- or a hygromycin-resistance DNA cassette (see below for their construction) as described (<http://podospora.igmors.u-psud.fr/>). The transformants were isolated on a regeneration medium contained saccharose (205g/L) and one or two of the following antibiotics: 75 μg/ml hygromycin (Roche), 50 μg/ml nourseothricin (Werner), 10 μg/ml phleomycin (Euromedex), or 0.5 μg/ml oligomycin (Sigma). For each transgenic strain, at least 3 clones corresponding to 3 independent chromosomal integrations that showed the same phenotype were retained. Chromosomal integration of the transgenes was verified by DNA sequencing. The selected clones were back-crossed to the wild type and segregation of the resistance marker in meiotic products was analyzed; all the analyzed clones exhibited a 2:2 segregation, indicative of a unique integration site. We additionally determined the levels of transcripts from the *Atp9* transgenes to exclude the possibility of multiple tandem repeat integrations.

**Obtainment of mutant strains of *P. anserina*.**

***^5^nat*.** We first constructed a *pPable* plasmid containing the coding sequence (CDS) of the *nat1* gene of *Streptomyces noursei* under the control of the 5’ and 3’ *cis-*regulatory sequences of *Atp9-5* (referred to as *^5^nat*). For this, the *nat1* CDS was PCR-amplified with primers 15 and 16 from the ClonNAT plasmid (Werner BioAgents) (see S5 Table for the sequences of the primers). The 5’ (585 nt) and 3’ (739 nt) regulatory sequences of *Atp9-5* were PCR-amplified from *S* wild type genomic DNA using primers 1+2 and 3+4, respectively. The three DNA fragments were mixed and PCR amplified with primers 1+4; the resulting PCR product was digested with *Hind*III *and Sal*I and cloned into *pPable* cut with the same enzymes. The wild type strain of *P. anserina* was transformed with the recombinant plasmid. Owing to the presence on this plasmid of the *ble* gene, *^5^nat* clones were selected for their ability to grow in the presence of phleomycin (PhleoR).

***^7^nat*.** We first constructed a *pPable* plasmid containing the CDS of *nat1* gene under the control of the 5’ and 3’ *cis-*regulatory sequences of *Atp9-7* (referred to as *^7^nat*). For this, the *nat1* CDS was PCR-amplified with primers 17 and 18 from the ClonNAT plasmid. The 5’ (1020 nt) and 3’ (670 nt) regulatory sequences of *Atp9-7* were PCR-amplified from *S* wild type genomic DNA using primers 5+6 and 7+8, respectively. The three DNA fragments obtained in this way were mixed and PCR amplified with primers 7+10. The resulting PCR product was digested with *Hind*III *and Sal*I and cloned into *pPable* cut with the same enzymes. The wild type strain of *P. anserina* was transformed with the recombinant plasmid and *^7^nat* clones were selected for their ability to grow in the presence of phleomycin (PhleoR).

***^Gpd^nat^AS1^***. Construction of this strain was described previously [1].

***^7^7[^5^5]^5^5***. We first constructed a pBChygro plasmid containing the wild type *Atp9-5* gene PCR amplified with primers 20 and 21. The PCR product contained the 585 nt upstream and 739 nt downstream the CDS of *Atp9-5*. It was digested with *Hind*III and *Not*I and cloned into the pBCHygro vector cut with the same enzymes. The plasmid harbors the *hph* gene that enabled selection on hygromycin plates of the *^7^7[^5^5]^5^5* clones after transformation of the wild type (*^7^7^5^5*) with this plasmid. The resulting *^7^7[^5^5]^5^5* strain was used to generate strains *[^7^5]^5^5* and  *^7^7[^5^5]^5^5_OR_* strains by genetic crosses.

***^7^7[^5^7]^5^5.*** Construction of this strain was described previously [2]. It expresses, in addition to the native *Atp9-7* and *Atp9-5* genes, an *Atp9-7* ectopic allele under control of the regulatory sequences of *Atp9-5* linked to a hygromycin-resistance cassette. The levels of transcripts from the modified *Atp9-7* gene (*^5^7*) were similar to those of the native *Atp9-5* gene (S2 Fig). This strain was used to generate strain *^7^7[^5^7]*.

***^7^7[^7^5]^5^5***. Construction of this strain was described previously [2]. It expresses, in addition to the native *Atp9-7* and *Atp9-5* genes, an *Atp9-5* ectopic allele under control of the regulatory sequences of *Atp9-7* linked to the phleomycin-resistance cassette (Phleo^R^). The levels of transcripts from the modified *Atp9-5* gene (*^7^5*) were similar to those of the native *Atp9-7* (S2 Fig.). This strain was used to generate strains *[^7^5]^5^5* and *^7^7[^7^5]^5^5_OR_* by genetic crosses.

***^5^5*.** Construction of this strain was described previously [2]. In this strain, the native *Atp9-7* gene has been deleted using a hygromycin-resistance DNA cassette. This strain was used to generate strains *[^7^5]^5^5* and *[^5^7]* by genetic crosses.

***^7^7[^5^5].*** This strain was obtained by deleting the native *Atp9-5* gene in the strain *^7^7[^5^5]^5^5* described above, using a nourseothricin-resistance (Nat^R^) cassette described in [2]. This strain had a wild type phenotype owing to the presence of an ectopic *Atp9-5* gene the transcriptional activity of which was similar to that of the native *Atp9-5* gene (S2 Fig.). This strain was used to generate strain *^7^7[^5^7]* by genetic cross.

***^7^7[^5^7]***. Construction of this strain was described in a previous study [2], in which it was referred to as Δ5 *7^5^*. It is deleted for *Atp9-5* and contains two *Atp9-7* genes, the native one and an ectopic version under control of the regulatory sequences of *Atp9-5*. It was obtained by crossing strains *^7^7^5^5[^5^7]* (Hygro^R^) and *^7^7[^5^5]* (Nat^R^ Hygro^R^) and selection in meiotic products of Hygro^R^ Nat^R^ clones. *^7^7^5^5* and *^7^7[^5^7]* progenies were distinguished by PCR analyses. The strain *^7^7[^5^7]* was used to generate strain *[^5^7]* by genetic cross and strain *^7^7[^5^7_OR_]* (see below).

***[^7^5]^5^5***. Construction of this strain was described in a present study [2], in which it was referred to as Δ7 *5^7^*. It is deleted for *Atp9-7* and contains two *Atp9-5* genes, the native one and the other under control of the regulatory sequences of *Atp9-7*. It was obtained by crossing the strains *^7^7[^7^5]^5^5* (Phleo^R^) and *^5^5* (Hygro^R^) and selection of Phleo^R^ Hygro^R^ meiotic progenies. The strain *[^7^5]^5^5* was used to generate strain *[^7^5] [^5^7]* by genetic cross.

***[^5^7]****.* Construction of this strain was described in a previous study [2], in which it was referred to as *Δ7 Δ5 7^5^* [2]. This strain is deleted for both the native *Atp9-5* and *Atp9-7* genes and expresses an ectopic version of *Atp9-7* under control of the regulatory sequences of *Atp9-5*. It was obtained by crossing strains *^5^5* (Hygro^R^) and *^7^7[^5^7]* (Nat^R^ Hygro^R^) and selection of Hygro^R^ Nat^R^ meiotic progenies. *^7^7[^5^7]* and *[^5^7]* progenies were distinguished by PCR analyses. The strain *[^5^7]* was used to generate strain *[^7^5][^5^7]* by genetic cross.

***[^7^5][^5^7]***. This strain is deleted for both the native *Atp9-5* and *Atp9-7* genes and expresses ectopic versions thereof in which *Atp9-5* is controlled by the regulatory sequences of *Atp9-7* and *vice versa*. It was obtained by crossing strains *^5^7* (Nat^R^ Hygro^R^) and *[^7^5]^5^5* (Phleo^R^ Hygro^R^) strains and selection of viable Phleo^R^ Hygro^R^ Nat^R^ meiotic progenies (the other possible Phleo^R^ Hygro^R^ Nat^R^ genotype (*^7^5*) is not viable).

***^7^7^5^5_OR_*.** Individual cultures of the wild type strain (*^7^7^5^5*) obtained after 48h of incubation on M2 medium from fresh pieces of young mycelium were covered with cellophane disk and exposed to UV light for 30 sec at 100 erg/s. After 24 h in the dark they were transferred to M2 medium supplemented with 3 μg/ml oligomycin. Only one O_R_ clone per culture was retained to ensure genetic independence of the isolates. All the analyzed clones showed a 2:2 segregation of oligomycin-resistance in meiotic products after crossing to the wild type. Three such clones were sequenced; they all carried a F124S substitution in the coding sequence of *Atp9-5*. This strain *^7^7^5^5_OR_* was used to generate strains *^7^7[^5^5]^5^5_OR_* and *^7^7[^7^5]^5^5_OR_* by genetic crosses.

***^7^7[^5^5]^5^5_OR_***. This strain was obtained by crossing strains *^7^7^5^5_OR_* (O_R_) and *^7^7[^5^5]^5^5* (Hygro^R^) strains and selection of O_R_ Hygro^R^ meiotic progenies.

***^7^7[^7^5]^5^5_OR_***. This strain was obtained by crossing strains *^7^7^5^5_OR_* (O_R_) and *^7^7[^7^5]^5^5* (Phleo^R^) and selection of O_R_ Phleo^R^ meiotic progenies.

***^7^7[^5^7_OR_]*.** This strain was obtained by UV irradiation of strain ***^7^7****[^5^7]*, using the same protocol used for selection of *^7^7^5^5_OR_* from the wild type strain *^7^7^5^5*.

**Longevity measurements**. These measurements were made in 30 cm long race tubes
containing M2 medium, from small pieces of young (one-day-old) mycelium derived from spores. The tubes were incubated at 27°C in the dark and examined daily to score the time at which the cultures died as manifested by the stop of growth and the appearance of a dark pigment in the apical cells. Longevity was expressed in centimetres of growth between the initiation of the culture (germinating filaments) and apical cell death. Life spans were also estimated by the number of days by which 50% of parallel cultures were still alive (half-life or median life span). For each genotype, the analysed spores were generated from different genetic crosses. For each cross, six spores were used of which three were in one of the two mating types. For each spore, three cultures were examined. The total number of cultures analysed for each genotype ranged from 32 to 196 (see S3 Table). A number of 30 independent cultures is considered to be sufficient to ascribe longevity values in *P. anserina* [3-5]. It is to be noted that the 'sister cultures' we made (max. three per spore) can be considered as biological replicates, according to previous work by Rizet [6] showing that longevity is in *P. anserina* ' determined' by an as-yet-unidentified cytoplasmic factor that randomly occurs during vegetative growth. Implants from 'determined' mycelium will produce cultures with the same longevity, and have therefore to be considered as 'technical replicates' or 'clones'. All our longevity measurements were performed from implants taken from young (one-day-old) not yet 'determined' mycelium. In that case, even when they originate from the same spore, implants can produce mycelia of different longevities, and can therefore be considered as biological replicates. Differences in longevity were statistically analyzed by student t-test. For life spans, statistics were made on parallel survival curves using a log rank test [7] (see Figure 3 and S3 Table).

**Preparation of protoplasts**. For each strain 20 bottles with 100 ml of MTR rich medium (50 g/L corn meal, 5 g/L yeast extract) were inoculated with small pieces of 2-day-old mycelium (scraped with a scalpel from 5-20 Petri dishes) that had been fragmented in a FastPrep apparatus (MP Biomedical) at speed 4 for 20 sec. The bottles were incubated for 48 hours (5 days in exceptional cases) without shaking in the dark. After growing, each culture was drained on two sheets of gauze and ~200 g of wet mycelium treated with 8 g Glucanex (Novozyms) in 200 ml of 0.6 M saccharose/ 5 mM Na2HPO4/ 45 mM KH2PO4 pH 5.5 for 3 hours at 37°C with slow agitation. Protoplasts were collected by filtration on 4 sheets of gauze and centrifugation at 2000g for 10 min.

1. El-Khoury R, Sellem CH, Coppin E, Boivin A, Maas MF, et al. (2008) Gene deletion and allelic replacement in the filamentous fungus Podospora anserina. Curr Genet 53: 249-258.

2. Déquard-Chablat M, Sellem CH, Golik P, Bidard F, Martos A, et al. (2011) Two Nuclear Life Cycle-Regulated Genes Encode Interchangeable Subunits c of Mitochondrial ATP Synthase in Podospora anserina. Mol Biol Evol 28: 2063-2075.

3. Knuppertz L, Hamann A, Pampaloni F, Stelzer E, Osiewacz HD (2014) Identification of autophagy as a longevity-assurance mechanism in the aging model Podospora anserina. Autophagy 10: 822-834.

4. Weil A, Luce K, Drose S, Wittig I, Brandt U, et al. (2011) Unmasking a temperature-dependent effect of the P. anserina i-AAA protease on aging and development. Cell Cycle 10: 4280-4290.

5. Scheckhuber CQ, Houthoofd K, Weil AC, Werner A, De Vreese A, et al. (2011) Alternative oxidase dependent respiration leads to an increased mitochondrial content in two long-lived mutants of the aging model Podospora anserina. PLoS One 6: e16620.

6. Rizet G (1953) [Longevity of strains of Podospora anserina]. C R Hebd Seances Acad Sci 237: 1106-1109.

7. Bland JM, Altman DG (2004) The logrank test. BMJ 328: 1073.
