# Supplementary material for: Regulation of Aerobic Energy Metabolism in Podospora anserina by Two Paralogous Genes Encoding Structurally Different c-Subunits of ATP Synthase
Source: PLoS Genet. 2016 Jul 21;12(7):e1006161. doi: 10.1371/journal.pgen.1006161 (PMC4956034; doi:10.1371/journal.pgen.1006161)
Supplement: S1 Table — For each strain, the allelic state of Atp9-7 and Atp9-5 and the presence of ectopic transgene(s) are presented. Superscripts denote the origin of the 5’ and 3’ cis-regulatory sequences (~500–600 nucleotides upstream the ATG for 5’ and ~600–800 nucleotides downstream the stop codon for 3’). Inactivated alleles (Δ) and transgenes are linked to an antibiotic-resistance cassette containing either hph (hygromycin-resistance), ble (phleomycin resistance) or nat1 (nourseothricin-resistance) genes. OR is for oligomycin-resistance conferred by the F124S or F135Y mutation in ATP9-5 and ATP9-7, respectively. (DOCX) [file pgen.1006161.s002.docx]

|  |  | *Genotype* | | |  | |  |
| --- | --- | --- | --- | --- | --- | --- | --- |
| *Strain name* | *Atp9-7* locus | | Ectopic | *Atp9-5* locus | | *Origin* | |
| *^7^7^5^5 (wt)* | *^7^7* | |  | *^5^5* | |  | |
| *^5^nat* | *^7^7* | | *^5^nat1::ble* | *^5^5* | | *this study* | |
| *^7^nat* | *^7^7* | | *^7^nat1::ble* | *^5^5* | | *this study* | |
| *^Gpd^nat^AS1^* | *^7^7* | | *^Gpd^nat1 ^AS1^* | *^5^5* | | *[1]* | |
| *^7^7[^5^5]^5^5* | *^7^7* | | *^5^5::hph* | *^5^5* | | *this study* | |
| *^7^7[^5^7]^5^5* | *^7^7* | | *^5^7::hph* | *^5^5* | | *this study* | |
| *^7^7[^7^5]^5^5* | *^7^7* | | *^7^5::ble* | *^5^5* | | *this study* | |
| *^5^5* | *Δ7::hph* | |  | *^5^5* | | *[2]* | |
| *^7^7[^5^5]* | *^7^7* | | *^5^5::hph* | *Δ5::nat* | | *this study* | |
| *^7^7[^5^7]* | *^7^7* | | *^5^7::hph* | *Δ5::nat* | | *[2]* | |
| *[^7^5]^5^5* | *Δ7::hph* | | *^7^5::ble* | *^5^5* | | *[2]* | |
| *[^5^7]* | *Δ7::hph* | | *^5^7::hph* | *Δ5::nat* | | *[2]* | |
| *[^7^5][^5^7]* | *Δ7::hph* | | *^7^5::ble ^5^7::hph* | *Δ5::nat* | | *[2]* | |
| *^7^7^5^5_OR_* | *^7^7* | |  | *^5^5_OR_* | | *this study* | |
| *^7^7[^5^5]^5^5_OR_* | *^7^7* | | *^5^5::hph* | *^5^5_OR_* | | *this study* | |
| *^7^7[^7^5]^5^5_OR_* | *^7^7* | | *^7^5::ble* | *^5^5_OR_* | | *this study* | |
| *^7^7[^5^7_OR_]* | *^7^7* | | *^5^7_OR_::hph* | *Δ5::nat* | | *this study* | |

**S1 Table. Strain genotypes**
