# Supplementary material for: Regulation of Aerobic Energy Metabolism in Podospora anserina by Two Paralogous Genes Encoding Structurally Different c-Subunits of ATP Synthase
Source: PLoS Genet. 2016 Jul 21;12(7):e1006161. doi: 10.1371/journal.pgen.1006161 (PMC4956034; doi:10.1371/journal.pgen.1006161)
Supplement: S2 Table — a Full genotypes of strains are given in S4 Table. b Germination efficiency in the absence of nourseothricin (nourseo) is expressed as the percentage of spores that germinated within a period of 7 days on G medium compared to the wild type strain. The germination efficiencies measured in the presence nourseothricin are for each strain expressed relative to those obtained without the drug. (DOCX) [file pgen.1006161.s003.docx]

| Strains ^a^ | Germination ^b^  efficiency | | Vegetative growth rate (mm/d) | |  | |
| --- | --- | --- | --- | --- | --- | --- |
|  | -nourseo | +nourseo | -nourseo | +nourseo | *n* |  |
| *wt* | 100 | 0 | 7.5±0.3 | 0 | 40 |  |
| *^5^nat* | 95±7 | 85±8 | 7.4±0.4 | 6.0±0.1 | 40 |  |
| *^7^nat* | 95±7 | 0 | 7.5±0.2 | 2.7±0.1 | 40 |  |
| *^Gpd^nat ^AS1^* | 95±7 | 90±6 | 7.4±0.5 | 6.5±0.2 | 40 |  |

**S2 Table. Phenotypes of the *nat* transgenic strains**
