# Supplementary material for: Regulation of Aerobic Energy Metabolism in Podospora anserina by Two Paralogous Genes Encoding Structurally Different c-Subunits of ATP Synthase
Source: PLoS Genet. 2016 Jul 21;12(7):e1006161. doi: 10.1371/journal.pgen.1006161 (PMC4956034; doi:10.1371/journal.pgen.1006161)
Supplement: S3 Table — a The full genotype of the strains is given in S2 Table. b Sporulation data were reported previously [7]: The efficiency of spore production is estimated counting the number of spores ejected in homozygous crosses. +++, ++, + and–correspond to 85–100%, 60–80%, 20–40% and less than 1% respectively. c Germination efficiency is expressed as the percentage of spores that germinated within a period of 7 days on G medium compared to the wild type strain. d Density of the mycelium (weight/area) was estimated on mycelium grown for 3 days. e Oligomycin resistance corresponds for each strain to the percentage of growth in presence of oligomycin compared to the growth without the drug (5–15 cultures). f Longevities are mean values estimated from at least 32 cultures for each analyzed strain, expressed in centimeters (cm) of growth reached when the strain died and the number of days (half-life or median life span) by which 50% of the cultures were still alive. nd: not determined, n: number of independent cultures. (DOCX) [file pgen.1006161.s004.docx]

| Early Development | | | | | |  | | | Vegetative growth | | | | | | | |
| --- | --- | --- | --- | --- | --- | --- | --- | --- | --- | --- | --- | --- | --- | --- | --- | --- |
| Strains^a^ | Spore^b^ | Germ^c^ | |  | | | Growth rate (mm/d) | | | Density^d^  (mg/cm^2^) | | Oligomycin  resistance^e^ | | Longevity^f^ | | |
|  |  |  |  |  | | |  |  |  |  |  |  |  | cm | *n* | Half life (d) |
| *^7^7^5^5* | +++ | | 100 | |  | | | 7.9 ± 0.4 | | | 11.5 | | 24 ± 4 | 9.4 ± 1.0 | 196 | 17.2 ± 2.7 |
| *^7^5[^5^7]* | +++ | | 95 ± 7 | |  | | | 7.7 ± 0.3 | | | 7.1 | | 22 ± 5 | 11.6 ± 0.9 | 49 | 20.1 ± 0.4 |
| *^5^5* | - | | 95 ± 7 | |  | | | 7.4 ± 0.2 | | | 7.6 | | 23 ± 4 | 7.4 ± 0.8 | 136 | 13.9 ± 1.1 |
| *[^7^5]^5^5* | ++ | | 95 ± 7 | |  | | | 7.3 ± 0.4 | | | 8.3 | | 24 ± 5 | 7.5 ± 0.8 | 57 | 15.8 ± 1.9 |
| *[^5^7]* | + | | 95 ± 7 | |  | | | 7.9 ± 0.2 | | | 9.3 | | 24 ± 6 | 12.1 ± 0.9 | 67 | 19.7 ± 1.7 |
| *^7^7[^5^7]* | +++ | | 95 ± 7 | |  | | | 7.5 ± 0.3 | | | 7.5 | | 23 ± 5 | 13.1 ± 1.5 | 72 | 21.3 ± 1.9 |
| *^7^7^5^5_OR_* | +++ | | 75 ± 5 | |  | | | 6.3 ± 0.5 | | | 4.0 | | 80 ± 6 | 9.9 ± 2.0 | 37 | 19.1 ± 3.5 |
| *^7^7[^5^7_OR_]* | +++ | | 75 ± 4 | |  | | | nd | | | nd | | 80 ± 4 | 9.1 ± 1.0 | 33 | 18.0 ± 3.3 |
| *^7^7[^5^5]^5^5_OR_* | +++ | | 40 ± 9 | |  | | | 7.6 ± 0.3 | | | 1.0 | | 33 ± 6 | 8.6 ± 1.2 | 40 | 20.2 ± 1.9 |
| *^7^7[^7^5]^5^5_OR_* | +++ | | 62 ± 9 | |  | | | nd | | | nd | | 73 ± 9 | 9.0 ± 1.0 | 32 | 17.1 ± 1.8 |

**S3 Table. Phenotypes of the *Atp9* mutant strains**
