# Supplementary material for: Regulation of Aerobic Energy Metabolism in Podospora anserina by Two Paralogous Genes Encoding Structurally Different c-Subunits of ATP Synthase
Source: PLoS Genet. 2016 Jul 21;12(7):e1006161. doi: 10.1371/journal.pgen.1006161 (PMC4956034; doi:10.1371/journal.pgen.1006161)
Supplement: S4 Table — MitoATP9-5 and MitoATP9-5 extracts were prepared from protoplasts of strains 55 and [57], respectively. Three independent experiments for each strain were performed. Oxygen consumption was determined using NADH as a respiratory substrate, alone (state) 4 or in the presence of ADP (state 3) and in the presence of CCCP (uncoupled respiration). The respiratory control ratio (RCR) corresponds to the ratio between state 3 and state 4 respiration rates. ATP/O is the number of ATP molecules formed per oxygen atom reduced. Mean data are given with standard deviation. (DOCX) [file pgen.1006161.s005.docx]

|  |  | Oxygen consumption  (nmol O.min^-1^.mg^-1^) | | |  | |  | |  | |
| --- | --- | --- | --- | --- | --- | --- | --- | --- | --- | --- |
| Sample | Assay | State 4  NADH | State 3  NADH+ADP | Uncoupled  NADH+CCCP | | RCR | | ATP Synthesis  (nmol ATP.min^1^.mg^1^) | ATP/O |  |
| Mito  ATP9-5 | A | 136 | 342 | 427 | | 2.5 | | 500 | 1.46 |  |
|  | B | 174 | 398 | 487 | | 2.3 | | 606 | 1.52 |  |
|  | C | 180 | 396 | 479 | | 2.2 | | 612 | 1.55 |  |
|  | mean | 163± 24 | 379± 32 | 464± 32 | | 2.3 | | 573 ± 63 | 1.51± 0.05 |  |
| Mito  ATP9-7 | A | 152 | 302 | 485 | | 2.0 | | 372 | 1.23 |  |
|  | B | 152 | 304 | 456 | | 2.0 | | 384 | 1.26 |  |
|  | D | 120 | 238 | 392 | | 2.0 | | 296 | 1.24 |  |
|  | mean | 141 ± 18 | 281 ± 37 | 444± 48 | | 2.0 | | 351± 48 | 1.25± 0.01 |  |

**S4 Table. Oxidative phosphorylation in MitoATP9-5 and MitoATP9-7.**
