# Supplementary material for: Regulation of Aerobic Energy Metabolism in Podospora anserina by Two Paralogous Genes Encoding Structurally Different c-Subunits of ATP Synthase
Source: PLoS Genet. 2016 Jul 21;12(7):e1006161. doi: 10.1371/journal.pgen.1006161 (PMC4956034; doi:10.1371/journal.pgen.1006161)
Supplement: S5 Table — Gene nomenclature is according to the Podospora anserina Genome Project (http://podospora.igmors.u-psud.fr/). Fw and Rev correspond to the couples of primers used for qRT-PCR experiments. In each couple of primers one (*) overlaps an exon-exon junction to minimize non specific amplification. (DOCX) [file pgen.1006161.s006.docx]

| \| Gene name \|  \| Primers sequence \| \|  \| \| --- \| --- \| --- \| --- \| --- \| \| Pa_5_9140 \| 1 \| GCATCACCTCTGCATACCCC \| \| \| \| *Atp9-5* \| 2 \| gtggtacCCATTGTGTCTGATGAGAAGG \| \|  \| \|  \| 3 \| ctgccccTAAGTGTTTTGCTTCGGAGGTC \| \|  \| \|  \| 4 \| ccccgtcgACCTCGAGTGAGGGCTGG \| \|  \| \|  \| 20 \| GGTTCGAACAAAACCAAGCTT \| \|  \| \|  \| 21 \| gggcggccGCATCAGGGTACCGGGAC \| \|  \| \|  \| Fw* \| TCATGGCCAAGTTCACCTAAGC \| \|  \| \|  \| Rev \| CGATCGCTCCCAGTGAGG \| \|  \| \| Pa_7_10 \| 5 \| ggggaagcTTGTTAGGAGAGGAGTGTACC \| \| \| \| *Atp9-7* \| 6 \| gtggtaccCATTTTGACGAGACGGAGAAAT \| \| \| \|  \| 7 \| tgcccctaaACATGTCGTCTGGGGCGCT \| \|  \| \|  \| 8 \| gggggtcGACCGCATCGATTGCGTGAC \| \|  \| \|  \| Fw* \| CGGCTCTCAGGTCCCAGC \| \|  \| \|  \| Rev \| AGTTTTGGACACTCAGAAATAC \| \|  \| \| *nat1* \| 15 \| cagacacaATGGGTACCACTCTTGACGAC \| \|  \| \|  \| 16 \| agcaaaacacTTAGGGGCAGGGCATGCTC \| \|  \| \|  \| 17 \| ctcgtcaaaATGGGTACCACTCTTGACGAC \| \|  \| \|  \| 18 \| agacgacatgtTTAGGGGCAGGGCATGCTC \| \|  \| \| Pa_3_1710 \| Fw* \| GATGTCTGTTCCCCATCGAC \| \|  \| \| *Aox* \| Rev \| GAGGAAATGTTGGCAGTGGTG \| \|  \| \| Pa_3_5410 \| Fw* \| AGTACGCTGAGTACATGCTC \| \|  \| \| *Gpd* \| Rev \| GGTGAAGACACCGGTGGAC \| \|  \| \| Pa_7_6690 \| Fw \| GGCTGGCTTGGCGATACC \| \|  \| \| *Pdf2* \| Rev* \| CTGGCCAAGGTCGAGATAG \|  \|  \| |
| --- | --- | --- | --- | --- | --- | --- | --- | --- | --- | --- | --- | --- | --- | --- | --- | --- | --- | --- | --- | --- | --- | --- | --- | --- | --- | --- | --- | --- | --- | --- | --- | --- | --- | --- | --- | --- | --- | --- | --- | --- | --- | --- | --- | --- | --- | --- | --- | --- | --- | --- | --- | --- | --- | --- | --- | --- | --- | --- | --- | --- | --- | --- | --- | --- | --- | --- | --- | --- | --- | --- | --- | --- | --- | --- | --- | --- | --- | --- | --- | --- | --- | --- | --- | --- | --- | --- | --- | --- | --- | --- | --- | --- | --- | --- | --- | --- | --- | --- | --- | --- | --- | --- | --- | --- | --- | --- | --- | --- | --- | --- | --- | --- | --- | --- | --- | --- | --- | --- | --- | --- | --- | --- | --- | --- | --- |

**S5 Table. List of primers.**
