# Supplementary figures and images for: Regulation of Aerobic Energy Metabolism in Podospora anserina by Two Paralogous Genes Encoding Structurally Different c-Subunits of ATP Synthase
Source: PLoS Genet. 2016 Jul 21;12(7):e1006161. doi: 10.1371/journal.pgen.1006161 (PMC4956034; doi:10.1371/journal.pgen.1006161)

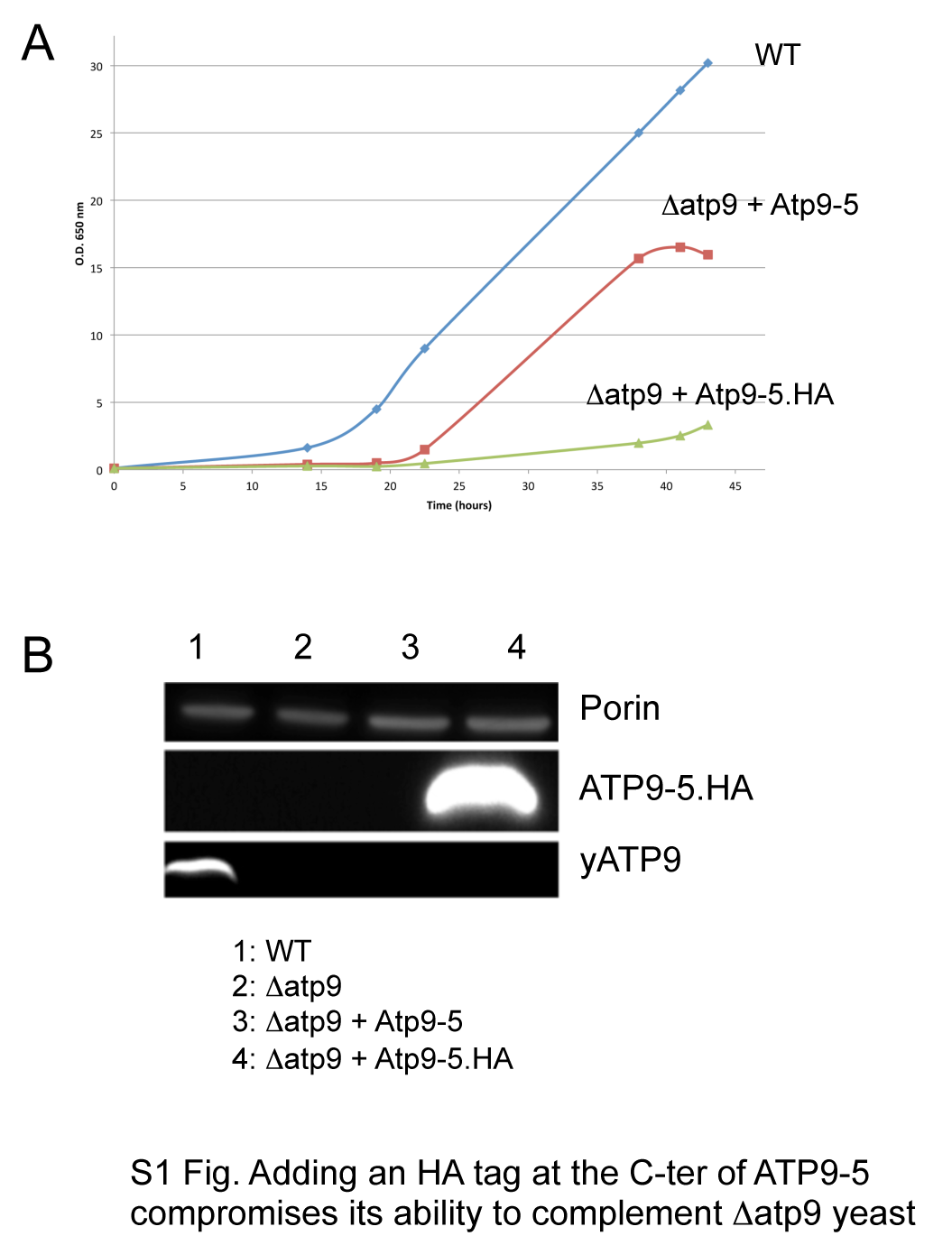

Supplement: S1 Fig — As we have shown previously [11], the Atp9-5 gene fused to a mitochondrial targeting sequence restores the capacity of a yeast strain lacking the mitochondrial ATP9 gene (Δatp9) to grow on respiratory substrate (glycerol). (A) Growth curves on glycerol showing that with an HA tag at its C-ter ATP9-5 can no longer restores mitochondrial function in Δatp9 yeast. (B) Western blots of total protein extracts with antibodies against porin, HA epitope, and yeast Atp9p (yATP9) showing ATP9-5.HA accumulation in Datp9 yeast transformed with the tagged gene. (TIF) [file pgen.1006161.s007.tif]

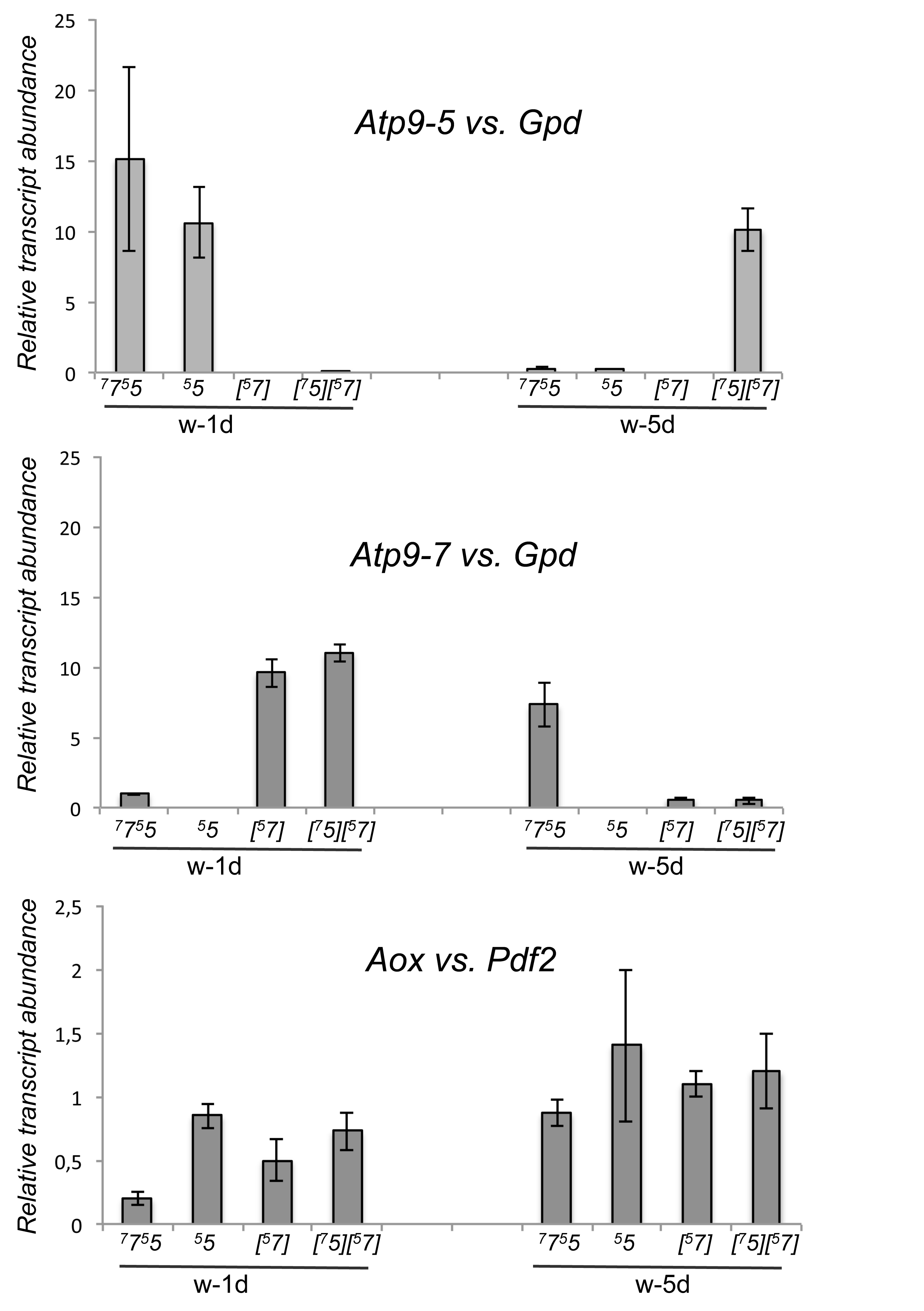


S2 Fig. Transcriptional activity of *Atp9-5*, *Atp9-7* and *Aox* in strains *7755* (*wt*), *55*, *[57]* and *[75][57]*.

Supplement: S2 Fig — RNA extracts were prepared from whole mycelium cultured on solid media for 2 days (w-2d), or 5 days (w-5d) (see Fig 1). The levels of mRNA transcripts from Atp9-5, Atp9-7 and AOX were determined by real-time quantitative reverse transcription PCR using, as indicated Gpd or Pdf2, as a reference gene. (DOCX) [file pgen.1006161.s008.docx]

**
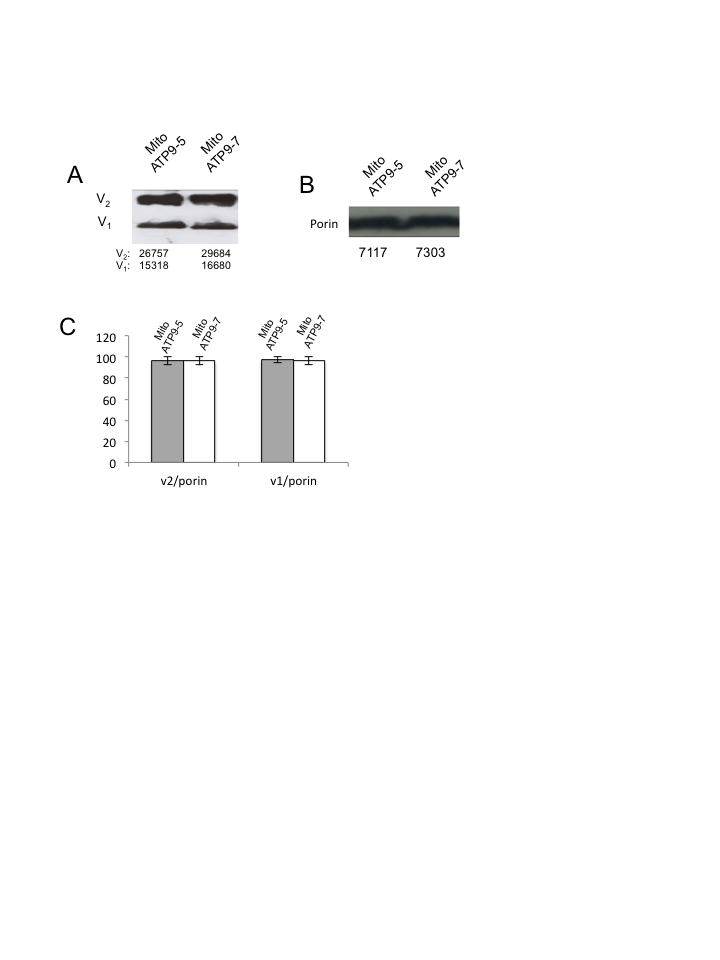
**

S4 Fig. Quantification of ATP synthase complexes in Mito-ATP9-5 and MitoATP9-7.

Supplement: S4 Fig — (A) BN-PAGE analysis of ATP synthase. On the left: Mito-ATP9-5 and MitoATP9-7 samples were extracted with 1% n dodecyl β D-maltoside (Sigma D4641), separated by BN-PAGE (50 μg per lane) and transferred to nitrocellulose membranes for Western blotting with antibodies against the yeast α-F1 protein. In the conditions used, ATP synthase was detected as dimeric (V2) and monomeric (V1) units. Below the blot are the results of the quantification of the immunological signals corresponding to V1 and V2 normalized to porin (see Materials and Methods). (B) 50 μg of proteins from MitoATP9-5 and MitoATP9-7 were separated via SDS-PAGE, transferred to a nitrocellulose membrane and probed with antibodies against porin. Below the blot are the results of the quantification of the immunological signals corresponding to porin (see Materials and Methods). (C): Mean values of the contents in V2 and V1 normalized to porin in the BN-PAGE experiments shown in Fig 4A and the one shown in panel A of this figure. (DOCX) [file pgen.1006161.s010.docx]
