# Supplementary material for: Regulation of Aerobic Energy Metabolism in Podospora anserina by Two Paralogous Genes Encoding Structurally Different c-Subunits of ATP Synthase
Source: PLoS Genet. 2016 Jul 21;12(7):e1006161. doi: 10.1371/journal.pgen.1006161 (PMC4956034; doi:10.1371/journal.pgen.1006161)
Supplement: S3 Fig — These measurements were made using 5-day-old mycelium (w-5d) of the wild type strain of P. anserina grown on solid plates. On the left (w-5d) are the results obtained with RNA extracts prepared from non-treated mycelium; on the right (w-5d gluc) are those obtained when RNAs were extracted from mycelium treated 3 hours in the presence of glucanex (conditions used to prepare protoplasts a-2d and a-5d in Fig 1). The constitutively expressed Gpd gene was used as a reference gene. (DOCX) [file pgen.1006161.s009.docx]

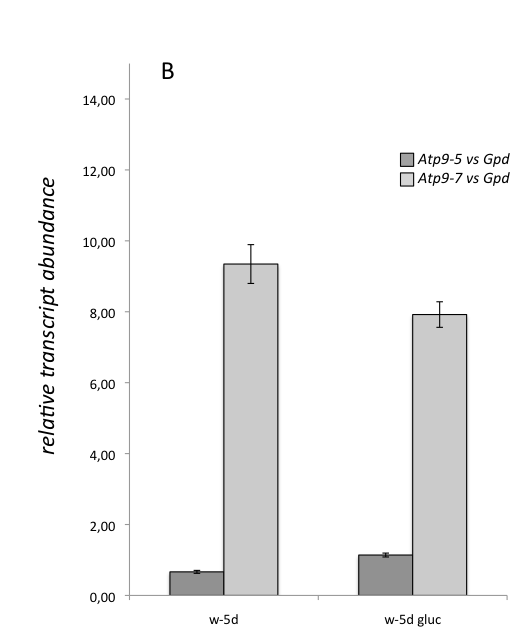


S3 Fig. Relative abundance of Atp9-5 and Atp9-7 transcripts in mycelium exposed or not to conditions used for preparing protoplasts from apical cells.
