# Supplementary material for: Regulation of Aerobic Energy Metabolism in Podospora anserina by Two Paralogous Genes Encoding Structurally Different c-Subunits of ATP Synthase
Source: PLoS Genet. 2016 Jul 21;12(7):e1006161. doi: 10.1371/journal.pgen.1006161 (PMC4956034; doi:10.1371/journal.pgen.1006161)
Supplement: S5 Fig — The shown alignment was established with ClustalW. The aligned protein sequences with a mitochondrial (mt) origin are from Homo sapiens (HOMSA), Bos Taurus (BOSTA), Drosophila melanogaster (DROME), Saccharomyces cerevisiae (SACCE), and P. anserina (PODATP9-5, PODATP9-7); those with a bacterial origin are from Bacillus pseudofirmus (BACPSOF4 and BACPSSP3), Ilyobacter tartaricus (ILYTA), Clostridium paradoxum (CLOPA), Synechoccus elongatus (SYNEL), Caldalkalibacillus thermarum (CALTH), Spirula platensis (SPIPL); the last sequence, from Spinacea oleracea (SPIOL), has a chloroplastic (chl) origin. The black arrows point to the Phenylalanine residues mutated in the oligomycin-resistant strains of P. anserina described in this study (F124S in ATP9-5; F135S in ATP9-7). The asterisk points to the essential Glutamate residue that is protonated/deprotonated during catalysis. Positions in the c-subunit, presumed to be important for the c-ring stoichiometry [27,44], are indicated by grey arrows. (DOCX) [file pgen.1006161.s011.docx]

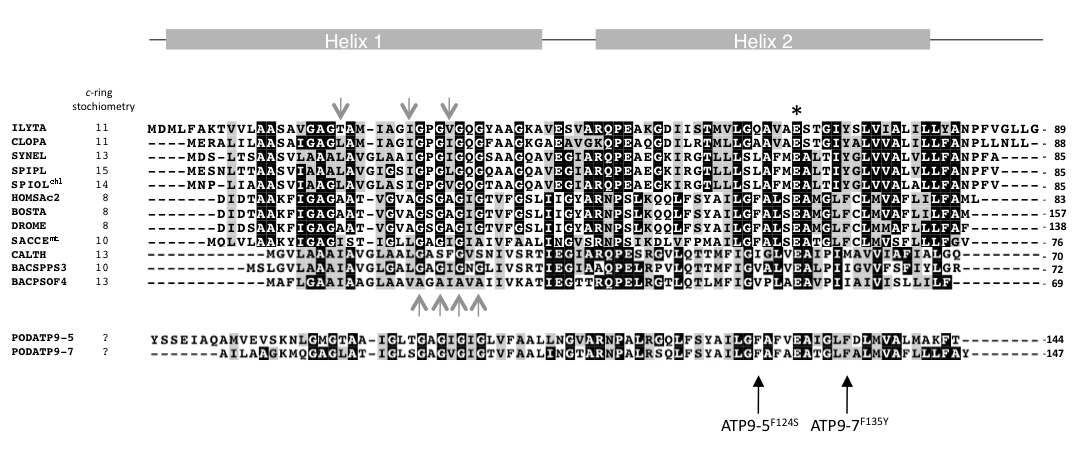


S5 Fig. Alignement of the proteins encoded by the *Atp9-7* and *Atp9-5* genes of *Podospora anserina* with *c*-subunits of known stoichiometry from various origins.
